# YB-1, an abundant core mRNA-binding protein, has the capacity to form an RNA nucleoprotein filament: a structural analysis

**DOI:** 10.1093/nar/gky1303

**Published:** 2019-01-03

**Authors:** Dmitry A Kretov, Marie-Jeanne Clément, Guillaume Lambert, Dominique Durand, Dmitry N Lyabin, Guillaume Bollot, Cyril Bauvais, Anastasiia Samsonova, Karina Budkina, Rachid C Maroun, Loic Hamon, Ahmed Bouhss, Ewen Lescop, Flavio Toma, Patrick A Curmi, Alexandre Maucuer, Lev P Ovchinnikov, David Pastré

**Affiliations:** 1 Institute of Protein Research, Russian Academy of Sciences, Pushchino 142290, Russian Federation; 2 SABNP, University of Evry, INSERM U1204, Université Paris-Saclay, 91025 Evry, France; 3 Institute for Integrative Biology of the Cell (I2BC), CEA, CNRS, Université Paris-Sud, Université Paris-Saclay, 91198 Gif-sur-Yvette, France; 4 Synsight, a/s IncubAlliance 86 rue de Paris Orsay 91400, France; 5 Institut de Chimie des Substances Naturelles, CNRS UPR 2301, Université Paris-Saclay, 91198 Gif sur Yvette cedex, France

## Abstract

The structural rearrangements accompanying mRNA during translation in mammalian cells remain poorly understood. Here, we discovered that YB-1 (YBX1), a major partner of mRNAs in the cytoplasm, forms a linear nucleoprotein filament with mRNA, when part of the YB-1 unstructured C-terminus has been truncated. YB-1 possesses a cold-shock domain (CSD), a remnant of bacterial cold shock proteins that have the ability to stimulate translation under the low temperatures through an RNA chaperone activity. The structure of the nucleoprotein filament indicates that the CSD of YB-1 preserved its chaperone activity also in eukaryotes and shows that mRNA is channeled between consecutive CSDs. The energy benefit needed for the formation of stable nucleoprotein filament relies on an electrostatic zipper mediated by positively charged amino acid residues in the YB-1 C-terminus. Thus, YB-1 displays a structural plasticity to unfold structured mRNAs into extended linear filaments. We anticipate that our findings will shed the light on the scanning of mRNAs by ribosomes during the initiation and elongation steps of mRNA translation.

## INTRODUCTION

After being exported from the nucleus to the cytoplasm, some mRNAs remain silent while others are rapidly engaged in translation. Controlling mRNA translation in cells contributes to the precise expression of genetic information in space and time (1,2). Decision about whether mRNA is going to be translated or repressed relies on the intricate parameters encompassing various factors binding to the 5′ and 3′ UTRs of mRNA (3,4), mRNA secondary structure (5–7) and RNA modifications (8). Moreover, even continuously translated mRNAs switch from time to time to an inactivate state as revealed by the stochastic pattern of single translation events in cells (9–11). Here we focus our attention on YB-1 (Y-box binding protein 1, YBX1), an abundant partner of mRNA in the cytoplasm (12,13), which was identified as a one of the major components of silenced messenger ribonucleoproteins (mRNPs) (14–16) and notably associated with the repressed paternal and maternal mRNA in spermatocytes and oocytes (17,18). YB-1 packages mRNA forming beads-on-a-string structures to repress translation *in vitro* (13,18,19). Sucrose gradient profiling also revealed the preferential presence of YB-1 in the non-polysomal fraction (20–22). While all these points are accepted, reducing YB-1 expression in myeloma cells (19) or suppressing the expression of Y-box proteins in *Caenorhabditis elegans* (20) affects the formation of polysomes, which might not have been expected for a pure translation repressor. In addition, YB-1 promotes the translation of some mRNAs such as *HIF-α* (21,22), *Snail1* (23) and *Rock1* (24). Melting of the secondary structure in 5′ UTR of *Snail1* and *HIF-α* mRNAs was suggested to explain the increased translation efficiency mediated by YB-1 (21,24), but no structural model was provided to explain mechanistically this hypothesis. How the packaging activity of YB-1 can be overcome in these conditions and how the unwinding is taking place remain unanswered questions. Some mRNA remodelers, such as eIF4A, an abundant mRNA helicase (25), may come into the play (26–28). Nevertheless, no protein factors have been reported yet to unfold mRNA packaged by YB-1.

We hypothesize that YB-1 packages mRNA to block translation and, under certain stimuli, undergoes a structural transition to unpack mRNA and promotes its translation. This hypothesis is based on two important considerations. First, YB-1 together with other Y-box binding proteins, YB-2 and YB-3, are major mRNA-binding proteins in the cytoplasm, that harbor a single cold-shock domain (CSD), a conserved five-strand *β*-barrel structure that binds to single stranded RNA/DNA (29). Y-box proteins are the closest mammalian cousins of cold shock proteins (CSPs) in bacteria and plants. CSPs are RNA chaperones (30,31) whose expression is dramatically upregulated in response to the low temperatures in order to melt cold-stabilized mRNA secondary structures and enable translation (32,33). Therefore, CSPs are positive regulators of translation in plants and bacteria (29). Second, YB-1 has also a long and unstructured C-terminal domain (CTD) that harbors many positively charged amino acid residues responsible for mRNA packaging *in vitro* (13). It is possible that phosphorylation taking place at identified sites in the CTD of YB-1 (34,35) may unlock translation repression exerted by the CTD.

**Figure 1.**
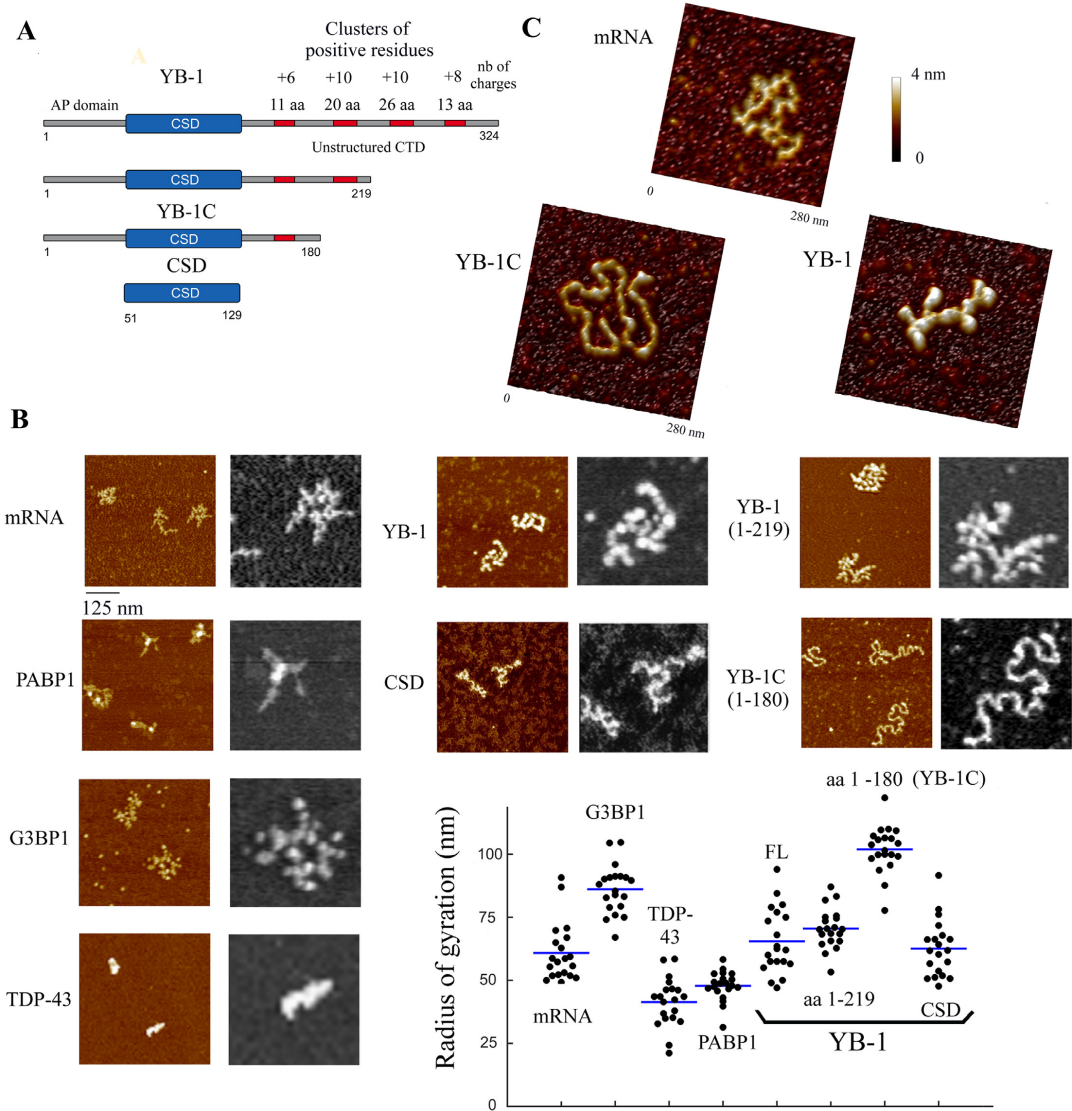
YB-1C, a truncated form of YB-1, forms a nucleoprotein filament with mRNA. (**A**) YB-1 constructs used in this study. The red boxes represent the positively charged residues located in the unstructured CTD. The residues are grouped in four clusters (see also supplementary Figure S10c). Only one positive cluster remains in YB-1C. AP domain: unstructured alanine and proline-rich domain. CSD: Cold-shock domain. Both the number of positive charges and amino acid residues (aa) contained in CTD clusters are indicated. (**B**) AFM images of mRNA:protein complexes deposited on mica. 2-Luciferase mRNA (3000 nt), 4 nM; PABP1, 1 μM; G3BP1, 2 μM; TDP-43, 0.5 μM; YB-1, 0.7 μM; aa 1–219, 1 μM; aa 1–180 (YB-1C), 2 μM; CSD, 10 μM. Lower right panel: scatter plot of the radii of gyration of mRNA:protein complexes. *n* = 20. Blue bar: mean. Only saturated mRNA:protein complexes are displayed (further increasing the concentration does not change the structure observed). (**C**) Zoom in on mRNA, the YB-1C nucleoprotein filament and YB-1-packaged mRNA, respectively.

To explore how YB-1 affects mRNA structure, we investigate by atomic force microscopy (AFM) the structural changes of nucleic acids induced by the binding of full length YB-1 or number of its mutants with truncated CTD. The results unravel the formation of extended nucleoprotein filaments when most of the positive residues of the CTD have been removed, with the exception of a cluster located about 20 residues away from CSD (Figure 1A). By combining nuclear magnetic resonance (NMR) spectroscopy, small-angle X-ray scattering (SAXS) and molecular dynamics (MD), we analyze the structure of the nucleoprotein filaments. We show that mRNA follows a helical path linking consecutive CSDs. About 6 nt per CSD are found in nucleoprotein filaments, revealing the dense packing of CSD along mRNA. The major driving force appears to be an electrostatic zipper formed by the positive charges located at the beginning of YB-1 CTD, which neutralizes negatively charged phosphates of the nucleotides interacting with an adjacent YB-1. Altogether, the results of this study introduce YB-1 as the first mRNA-binding protein with the capacity to form nucleoprotein filaments with mRNA. The formation of linear mRNA nucleoprotein filaments by YB-1 may play a key role in the handling of mRNA by large molecular machineries associated with the initiation and elongation steps of translation. Indeed, both the secondary structure of mRNA and the short persistence length of single-stranded RNA could be barrier for the scanning by ribosomes. These findings may provide a new perspective to unravel how mRNA can switch from inactivate to active state by disentangling the branched structure of mRNA and stabilizing it in a linear conformation. The present study is focused on the structural plasticity of mRNA while interacting with YB-1. Future investigations will probe whether the formation of mRNA nucleoprotein filaments reported here takes place in a cellular context and whether the formation of mRNA nucleoprotein filaments is critical for mRNA translation in the cellular context.

## MATERIALS AND METHODS

### AFM imaging and image analysis

AFM images were recorded in air by using a Nanoscope V Multimode 8 (Bruker, Santa Barbara, CA, USA) in PeakForce Tapping (PFT) mode using Scanasyst-Air probes (Bruker). Continuous force–distance curves were thus recorded with an amplitude of 100–300 nm at low frequency (1–2 kHz). PFT mode decreases the lateral and shear forces. Images were recorded at 2048 × 2048 pixels at a line rate of 1.5 Hz. Images shown in the figures are representative of three different and independent samples. The ‘particle analysis’ tool in the Nanoscope Analysis software (version 1.50) was used to determine the heights of the adsorbed mRNPs from at least three independent samples. Basically, for each particle of interest, the particle analysis tool measured the maximum height of the particle. The length of nucleoprotein filaments was measured by using the ImageJ software manually.

Indicated amounts of proteins and nucleic acids were incubated in 20 μl of binding buffer (10 mM Hepes, pH 7.5, 30 mM KCl, 1 mM Putrescine) for 5 min at room temperature. Putrescine, a natural polyamine, was added to the buffer to adsorb mRNPs on mica (36). Then, samples were deposited on the mica surface and dried. The method is described in details in ref. (36).

### NMR analysis

All NMR experiments were performed on 60 μl samples prepared in 50 mM potassium phosphate buffer pH 6.8, using 1.7 mm diameter capillary tubes. Control with additional salt (+100 mM KCl) gives similar results for YB-1C interaction with 30-nt long poly-(dC) ssDNA (data not shown).

NMR spectra of ^15^N-labeled CSD and YB-1C, free and in interaction with homo-DNA oligonucleotides, were acquired at 298 K on a Bruker AVIII HD 600 MHz spectrometer equipped with triple-resonance cryoprobe. Interaction of YB-1C with homo-RNA oligonucleotides was explored at 298 and 303 K.

YB-1C resonance assignments were achieved using 2D ^1^H-^15^N HSQC, 3D HNCO, 3D HNCA, 3D HN(CO)CA, 3D HNCACB, 3D HN(CO)CACB and 3D NOESY ^1^H-^15^N-HSQC experiments recorded at 298K on a Bruker 950 MHz spectrometer (TGIR, Gif-sur-Yvette and Grenoble, France) equipped with triple-resonance cryoprobe, using 1 mM [U-^15^N,^13^C] samples.

The binding of CSD or YB-1C to DNA or RNA was investigated using 2D ^1^H-^15^N HSQC recorded on 100 μM protein samples in presence of homo-DNA or -RNA oligonucleotides (C, T or U) with different size (5, 10, 20 or 30 nt) at a 1:1 molar ratio. Paramagnetic relaxation enhancement (PRE) experiments were recorded on MTSL-labeled (T62C, T89C, T108C, V114C, E117C) YB-1C mutant samples using the same conditions. For the inter-molecular PRE measurements, NMR samples contained 50 μM ^15^N-labeled YB-1C, 50 μM MTSL-labeled (T62C or V114C) YB-1C mutant and 100 μM dC30.

2,2- Dimethyl-2-silapentane-5-sulfonic acid was used as an external reference in pure D_2_O for chemical shift referencing. Data were processed and analyzed using Topspin 3.5 (Bruker) and CcpNmr Analysis 2.4.1 software (37).

Assignments: For isolated CSD, we assigned the amide resonances of 79% of residues (61/77 non-proline residues). Indeed, loop residue (93–104) signals could not be detected. Concerning YB-1C, we assigned about 90% of N-terminal and CSD residues (37/42 and 70/77 non-proline residues for N-terminal domain and CSD, respectively), and 79% of CTD residues (38/48 non-proline residues). The amide resonances from some residues could be observed only in presence of the dC10 homo-oligonucleotide.

### Small angle X-ray scattering (SAXS)

SAXS measurements were performed on the BM29 beamline at the ESRF (Grenoble, France) (38). The X-ray wavelength was equal to 0.992 Å and the sample to detector distance to 2.867 m, leading to an accessible q-range [0.004 Å^−1^, 0.5 Å^−1^] with *q* as the momentum transfer (*q* = 4π sin θ/λ) and 2θ the scattering angle. A 50 μl aliquot of sample was flowing through quartz capillary (1.8 mm in diameter) with a speed adapted to record 10 × 1 s frames. The online data reduction and analysis software (39) checks the perfect similarity of these frames and only keeps those frames that are strictly identical, ensuring that the final averaged pattern is free from the effects of any potential radiation damage.

All scattering curves I(q) were then processed using the program package PRIMUS/qt (40). The scattering intensity at the origin I (0) and R_g_ were evaluated using the Guinier approximation (41). P(r) profiles were determined using the indirect Fourier transform method as implemented in the program GNOM, yielding the value of the maximum diameter *D*_max_. The averaged molecular weight of the scattering objects was obtained by combining the different methods proposed in PRIMUS/qt. This allowed the stoichiometry of the YB-1C:ssDNA complexes to be determined.

The low-resolution shapes of these complexes have been restored by using an *ab initio* method. Here the complex is described as a compact ensemble of spheres, termed dummy atoms, the scattering of which is as close as possible to the experimental curve. The program MONSA (42) attributes different electronic densities to YB-1C spheres and to ssDNA spheres, making it possible to obtain the envelope of each phase (protein or DNA) within the complex.

### Molecular modeling

**Figure 2.**
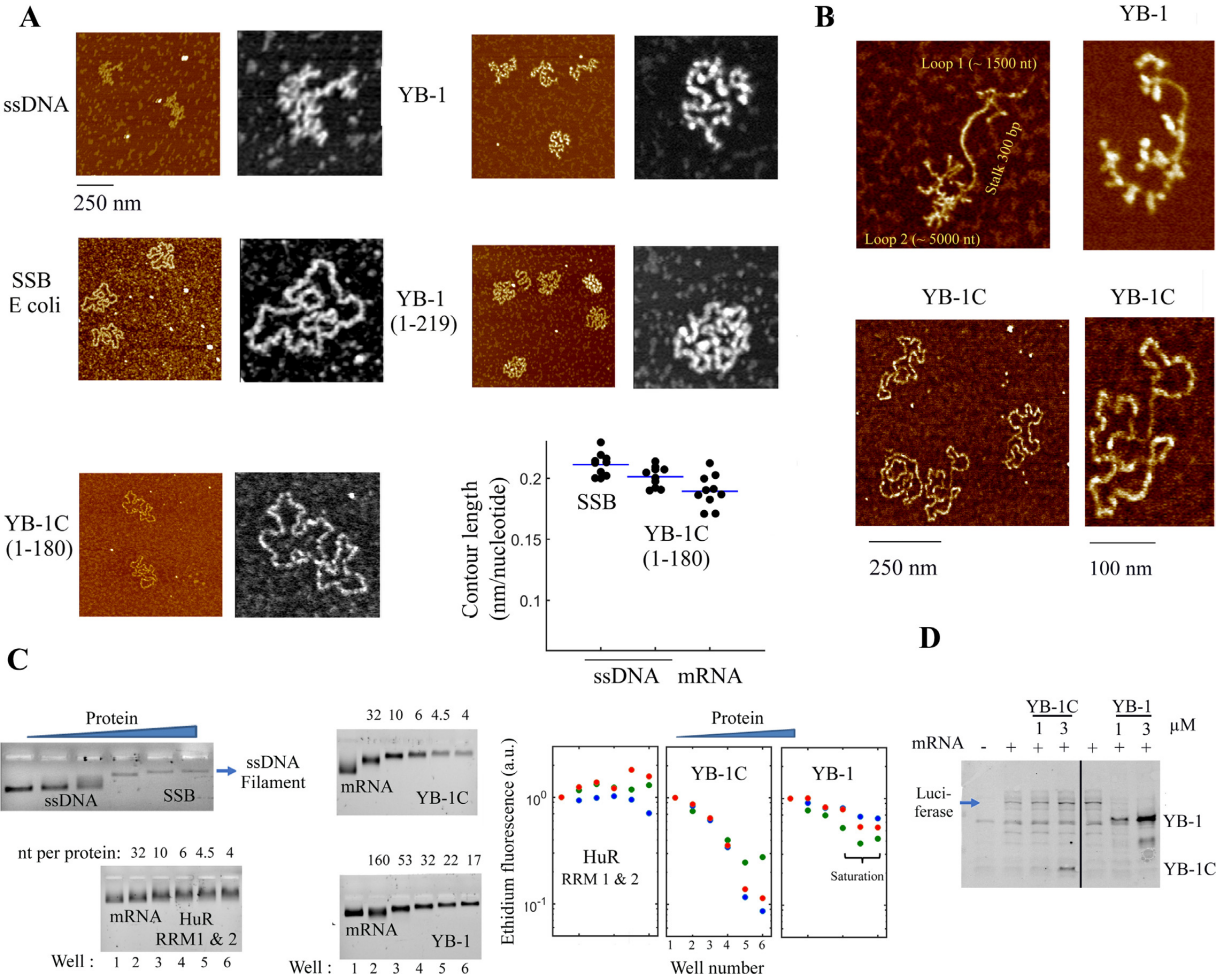
YB-1C also forms ssDNA nucleoprotein filaments, unwinds secondary structures and is compatible with mRNA translation. (**A**) AFM images of circular ssDNA (M13) complexed with indicated proteins at saturation. M13 ssDNA, 2 nM; SSB, 1 μM; YB-1, 0.5 μM; aa 1–219, 0.7 μM; aa 1–180 (YB-1C), 2 μM. Lower right panel: contour length of ssDNA or mRNA nucleoprotein filaments. *N* = 10. Blue bar: mean. (**B**) A DNA construct consisting of two circular ssDNA (1500 and 5000 nt) separated by a dsDNA stalk (300 bp) complexed with either YB-1 or YB-1C. ssDNA, 0.5 nM; YB-1, 0.5 μM; aa 1–180 (YB-1C), 2 μM. (**C**) Left panel: electrophoretic mobility of mRNA or ssDNA in the presence of different proteins. The presence of YB-1C at saturation decreases EtBr fluorescence of the mRNA band, as observed for ssDNA nucleoprotein filament with *Escherichia coli* SSB. Right panel: quantification of ethidium fluorescence of three replicates under the same condition. (**D**) *In vitro* translation assay in RRL. mRNA was pre-incubated with proteins for 10 min before addition in RRL for 10 min. Note the inhibition of mRNA translation by YB-1 but not by YB-1C. mRNA: 4 nM. Anti-YB-1 and anti-luciferase primary antibodies. Both endogenous and overexpressed YB-1 constructs are recognized by the anti-YB-1 antibody (see also Supplementary Figure S1).

Three-dimensional (3D) models of the trimer complexes YB-1:RNA and YB-1:DNA were considered for MD simulation as described in the simulation protocol. Each YB-1 trimer complex is composed of two YB-1 (48–165 positions), one C-terminus-truncated YB-1 (48–129 positions) located at the edge of the complex and 16 cytosine nucleotides (RNA or DNA). This system corresponds to the repeated motifs that may be present in the linear nucleoprotein filament. The only human wild-type YB-1 structure available has been resolved by NMR (position 52–129 PDB code: 1H95) (43). This NMR structure was used as a starting building block for the cold shock domain to set the YB-1 model. The 48–51 and 130–165 positions have been completed as unstructured protein conformation. The N-terminus of YB-1, aa 1–48, was removed according to the absence of interaction of this domain with either long or short DNA/RNA oligonucleotides. A crystal structure of bacterial cold shock proteins (CspB) has been resolved with a resolution of 1.68 Å with an uracil hexanucleotides (PDB code: 3PF5) (44). The *Bacillus subtilis* Bs–CspB complex has been used to set the position and the orientation for nucleotide fragment by aligning YB-1 structure to bacterial CspB. Then, the four uracil nucleotides have been replaced by cytosine, which represents the predicted number of nucleotides per CSD considering pi-pi stacking pairings (44). An additional 2-nt linker has been added to join the RNA or DNA backbone to the next YB-1 CSD. This number of 6 bases is consistent with experimental SAXS results (Figure 2D). The appropriate top to tail orientation about the next YB-1 has been set to avoid clashes and close contacts between CSDs. Then, the CTD region has been set as an unstructured conformation and accommodated to form positive electrostatic proximity between phosphate backbone (nucleic acid) and the arginine-rich region, according to NMR data (Figure 4B). To limit perturbation from a free CTD within the trimer complex, the CTD of the last YB1 has been truncated. However, we can see that we have still boundary issues. For instance, the basic CTD of the YB-1 protein located in the middle of the trimers partly disrupts interactions of RNA with conserve aromatic residues of its own CSD, most probably because it has not been deployed properly to the nearby CSD. To solve this problem, conserved interaction with RNA/DNA can be fixed (less realistic) or a longer oligomer has to be considered (more demanding in terms of computing time).

### Simulation protocol

All MD simulations have been carried out with GROMACS software package version 5.4.1 (45) using the ff03 amber force field with associated nucleic acid parameters (46) and TIP3P water model (47) to model interactions between protein, RNA/DNA and water. Each complex, YB-1-RNA or YB-1-DNA was put at the center of a cubic box with at least 1.0 nm distance between the boundary of the box and the protein–nucleic acid complex and neutralized by adding five chloride anions. The box was filled with water molecules. The total number of atoms for YB-1-RNA and YB-1-DNA are 126–115 and 126–102, respectively. Periodic boundary conditions are used, and the electrostatic interactions are calculated via the PME algorithm (48). To avoid steric clashes during solvation and relating to the size of the system, the system was relaxed first by steepest descent energy minimization. A 1-ns MD simulation using NVT protocol has been performed by imposing position restraints about 1000 kcal/mol only on protein and nucleic acid. An additional 1-ns MD simulation using NPT protocol has been done by applying 1000 kcal/mol position restraints on the protein and nucleic acid. Then two MD simulations were performed during 5 ns each to gradually relax the system by imposing position restraints only to nucleic acid, about 100 kcal/mol and 10 kcal/mol forces, respectively. For integration of the equations of motion, a 2 fs time step is used, with hydrogen atoms constrained by the LINC algorithm (49). The temperature of the system is kept at 300 K by the Parrinello−Donadio−Bussi thermostat (*τ* = 0.1 fs) (50) and the pressure at 1 bar by Parrinello−Rahman algorithm (*τ* = 2 fs) (51).

Using the NPT protocol where the system was totally free to move, an equilibration of 100 ns, followed by 300 ns for production were performed. The last geometry of the production phase has been energy-minimized using the steepest descent algorithm. The RMSD of backbone atoms for protein and nucleic acids have been measured using tools of GROMACS and plotted in Supplementary Figure S5, which corresponds to the last 100 ns of production phase. The RMSD variations do not exceed 0.5 nm and represent a stable state for each system. Trajectories and geometries have been visualized and represented by using VMD (52) and Swiss-Pdb Viewer (53). The MD simulations in this work were done using NVIDIA GPU resources owned by Synsight.

### Plasmids construction and protein overexpression

The complete coding region of *Homo Sapiens* YB-1 was optimized for *Escherichia coli* expression and cloned into pET22b expression vector at NdeI/XhoI sites. The 1–180 sequence of YB-1 (YB-1C) was amplified from the pET22b-YB-1 where sites for NdeI/XhoI were incorporated into the primers and cloned into pET22b.

The constructed plasmids were transformed into BL21 (DE3) cells and grown at 37°C in 2YT medium (non-labeled proteins) or in minimal medium M9 supplemented with 15N or/and 13C to obtained labeled proteins. The expression of the proteins was induced by IPTG 1mM added at OD600 = 0.7. The culture was then incubated for additional 4 h at 37°C. Cells were harvested by centrifugation at 2000 × *g* for 20 min at 4°C.

Recombinant *E. coli* single stranded DNA binding (SSB) protein was purchased from Abcam (ab123224).

HuR (RRM 1 and 2, aa 1–188) fragment corresponds to N-terminal fragment of human HuR protein (ELAVL1). The DNA, encoding this protein fragment was cloned in pET28b plasmid. PAPB1 and TDP-43 were produced and expressed as previously described (54,55).

### Protein purification

His-tagged proteins were purified under native conditions. Briefly, cells were harvested and resuspended in 20 ml of lysis buffer (20 mM Tris–HCl, pH 7.6, 10 mM imidazole, 2M KCl, 0.5 mM DTT, protease inhibitor tables (Roche)) and were lysed by sonication. The lysate was cleared by centrifugation at 70 000 × *g* for 1 h at 4°C. The supernatant was added to Ni-NTA agarose slurry for 30 min at 4°C under agitation and then loaded onto a gravity column. The column was washed with buffer (20 mM Tris–HCl, pH 7.6, 10 mM imidazole, 500 mM KCl, 0.5 mM DTT, 0.5 mM PMSF) and eluted with the same buffer supplemented with 250 mM imidazole. The eluate was then dialyzed against 20 mM Tris–HCl, pH 7.6, 500 mM KCl. Then RNase A treatment was applied to the samples to remove all traces of RNA contaminants for 1 h at room temperature under agitation. A second purification step was performed as described above. Then, the eluate was dialyzed against 50 mM phosphate buffer, pH 6.8, 500 mM KCl for YB-1C or 1M KCl for full length YB-1 and concentrated with Spin-X UF concentrators (Corning).

Non-tagged YB-1C proteins were purified by chromatography on Heparin–Sepharose 4B and Superose 12 HR 10/30 columns (Amersham Biosciences) as previously described (56). We then compared the structure of His-tagged and non his-tagged YB-1C with or without DNA oligonucleotides and observed no significant changes between the two proteins (data not shown).

### MTSL labeling

Mutated forms of YB-1C used for MTSL labeling by replacing the target amino acid by cysteine at located sites were made using the QuikChange II Site-Directed Mutagenesis Kit (Agilent technologies). The mutated proteins were then expressed as for YB-1C form and dialyzed against 20 mM phosphate buffer, pH 7.6, 500 mM KCl. Then, 2 mM DTT was added to the proteins in order to completely reduce oxidized thiol groups for 2 h at 4°C. PD-10 column (GE Healthcare) was used to remove the reducing agent and MTSL label (1-oxyl-2,2,5,5-tetramethyl-2,5-dihydro-1H-pyrrol-3-yl)methyl methanesulfonothioate) was added at 10-fold excess to the proteins. The proteins were incubated with MTSL label overnight at 4°C under agitation. PD-10 column was used to remove excess MTSL labeling and then the labeled proteins were dialyzed against 50mM phosphate buffer, pH 6.8, 500 mM KCl and concentrated with Spin-X UF concentrators (Corning).

### Gels mobility shift assays

Indicated amounts of YB-1, YB-1C and HuR (RRM 1&2) were incubated with 0.16 pmol of *2Luc* mRNA in 20 μl of binding buffer (20 mM Hepes, pH 7.5, 40 mM KCl) at room temperature for 5 min. For SSB mobility shifts assays, 67 fmol of M13 ssDNA (Biolabs) was incubated with indicated amounts of SSB protein in 20 μl of binding buffer (20 mM Tris–HCl, pH 7.6, 300 mM KCl) at 37°C for 15 min. Complexes were separated in 0.65% agarose gel in 0.5× TAE buffer at 5 V/cm for 45 min and were stained with 0.5 μg/ml ethidium bromide.

### *In vitro* translation assays

Translation occured in an incubation mixture (25 μl) containing 17.5 μl of nuclease-treated rabbit reticulocyte lysate (RRL) (Promega), amino acids (20 μM), buffer (10 mM Hepes, pH 7.5, 20 mM KCl), 200 ng of *Luciferase* mRNA (Promega) and YB-1 or YB-1C proteins at indicated concentrations. *Luciferase* mRNA was capped using Vaccinia Capping System according to the manufacturer’s recommendations (NEB). Translation was performed at 30°C for 10 min and 5 μl of the translation reaction was loaded onto 10% acrylamide gel. Proteins produced during the reaction and control proteins were visualized by western-blotting using specific antibody (anti-Luciferase (Sigma catalog number), anti-YB-1 (59-Q, Santa Cruz, epitope: aa 51–140), anti_RPL10A (Abcam ab187998). The DNA construct (57) consisting in two ssDNA loop (5000 and 1500 nt) separated by a dsDNA (300 bp) stalk is a gift from Dr Enrique Viguera Mínguez.

## RESULTS

### YB-1C forms a linear nucleoprotein filament with mRNA or ssDNA

Previous structural analyses have revealed the packaging of mRNA by YB-1 into beads-on–a-string structures at high protein:RNA ratio (56,58). YB-1 CTD is intrinsically unstructured and contains four clusters of positively charged residues that may have the capacity to compact mRNA through electrostatic interactions. To probe this point, we cloned several truncated YB-1 mutants where the positive charges in the unstructured CTD were progressively removed (Figure 1A). The structures of ribonucleoproteins (mRNPs) preformed in the presence of saturating amounts of various RNA-binding proteins and 2-*Luciferase* mRNA (56), considered as a model mRNA, were then analyzed and compared by high resolution AFM (Figure 1B and C). Surprisingly, we discovered that YB-1C (aa 1–180), a truncated form of YB-1, which contains a single cluster of six positively charged amino acid residues in CTD (aa 146–156) forms long and linear nucleoprotein filaments with mRNA. None of the other RNA-binding proteins tested, G3BP1, TDP-43 and PABP1 led to the formation of similar nucleoprotein filaments. The diameter of the nucleoprotein filament is about 3 nm what most probably corresponds to a single layer of YB-1C oligomer along mRNA (Figure 1C), because the diameter of CSD barrel is about 2 nm. As CSD binds to RNA but also ssDNA through the base stacking with conserved aromatic residues without significant differences (43,44), we then wondered whether YB-1C could also form a nucleoprotein filament with ssDNA. Again, YB-1C has the ability to unfold structured M13 circular ssDNA to form a circular nucleoprotein filament, in a way similar to *E. coli* SSB protein (36) (Figure 2A). A construct possessing two long ssDNA loops separated by a dsDNA stalk further showed the capacity of YB-1C to form a filament with ssDNA regions while avoiding the interaction with dsDNA stalk (Figure 2B). The lengths of mRNA and ssDNA nucleoprotein filaments are about 19 and 20 nm per 100 nt, respectively (Figure 2A). Therefore, mRNA or ssDNA nucleoprotein filaments formed with YB-1C probably share the similar structure.

Increasing the length of the YB-1C CTD by 39 additional amino acids (aa 1–219) including 10 arginine residues prevents the formation of linear nucleoprotein filaments (Figures 1B and 2A). However, we noticed a partially filamentous structure with ssDNA (Figure 2A) and a decrease in mRNA packaging compared to full length YB-1 (Figure 1B). Thus, the absence of two clusters of positively charged residues at the extremity of the CTD has partly diminished mRNA packaging activity of YB-1. We then considered whether CSD alone was sufficient to form a nucleoprotein filament with mRNA (Figure 1B). Even at high CSD concentrations (up to 100 μM), we failed to observed any mRNA filamentous structure. The CSD of YB-1 alone has a low affinity for RNA/ssDNA since the clusters of positive residues in the YB-1 CTD are responsible for the high affinity of YB-1 for nucleic acids (43). Accordingly, in bacteria and plants, CSD chaperone activity requires an elevated concentration of CSPs (33). The unstructured domains flanking CSD (aa, 1–51 and/or 130–180) may therefore play a key role in the formation of the nucleoprotein filament by YB-1C.

The linearization of the otherwise branched secondary structure of mRNA, orchestrated by YB-1C, requires the unwinding of mRNA secondary structure, at least partially. To probe the presence of mRNA secondary structure in the nucleoprotein filament, we analyzed the fluorescence of ethidium bromide (EtBr) in RNA:protein complexes. Given the high-fluorescence yield of EtBr intercalated in double stranded nucleic acids, melting mRNA secondary structures should reduce EtBr fluorescence. To control this point in the context of nucleoprotein filament formation, we show that SSB at saturation significantly reduces the intensity of EtBr fluorescence of ssDNA nucleoprotein filaments (Figure 2C). In agreement with the linearization of branched mRNA by YB-1C, a marked reduction in EtBr fluorescence is detected in YB-1C:mRNA complexes at saturation (about 4.5 nt per YB-1C, molar ratio). In contrast, elevated concentrations of two RRMs (RNA-Recognition Motifs, here from HuR), a classical RNA-binding domain or YB-1 at saturating concentration (20 nt per YB-1) failed to disrupt the secondary structure to the same extent (Figure 2C). However, a moderate decrease in EtBr fluorescence with YB-1 at saturation indicates a tendency to disrupt secondary structure, as previously noticed (59,60).

Then we wondered whether mRNA nucleoprotein filaments could be recognized by translation initiation factors and ribosomes to synthetize proteins. To explore this point, mRNA encoding Firefly luciferase was complexed with YB-1 or YB-1C (Figure 2D; Supplementary Figure S1a and b). YB-1 strongly inhibits translation of *Firefly luciferase* mRNA *in vitro*, as previously reported (13). On the other hand, translation efficiency was preserved in the presence of YB-1C. Therefore, mRNA nucleoprotein filaments are compatible with mRNA translation. Interestingly, translation efficiency even slightly increases when mRNA was premixed with YB-1C as compared to the addition of YB-1C after mRNA to rabbit reticulocyte lysate (Supplementary Figure S1c), indicating that formation of mRNA nucleoprotein filaments could be advantageous for efficient translation.

### YB-1C oligomerizes with 30 nt-long nucleic acids

To unravel the mechanism responsible for the formation of YB-1C nucleoprotein filaments with single stranded nucleic acids, we analyzed the structure of YB-1C:DNA or YB-1C:RNA complexes in liquid by NMR spectroscopy. We anticipated that the interaction of YB-1C with sufficiently long oligonucleotides would produce a short mimic of the nucleoprotein filaments detected by AFM (Figure 1B). Either full length YB-1 or YB-1_1–219_ were not amenable to NMR analysis (Supplementary Figure S2a), possibly due to multivalent interactions between acidic and basic domains located in the C-terminus of YB-1 (56).

**Figure 3.**
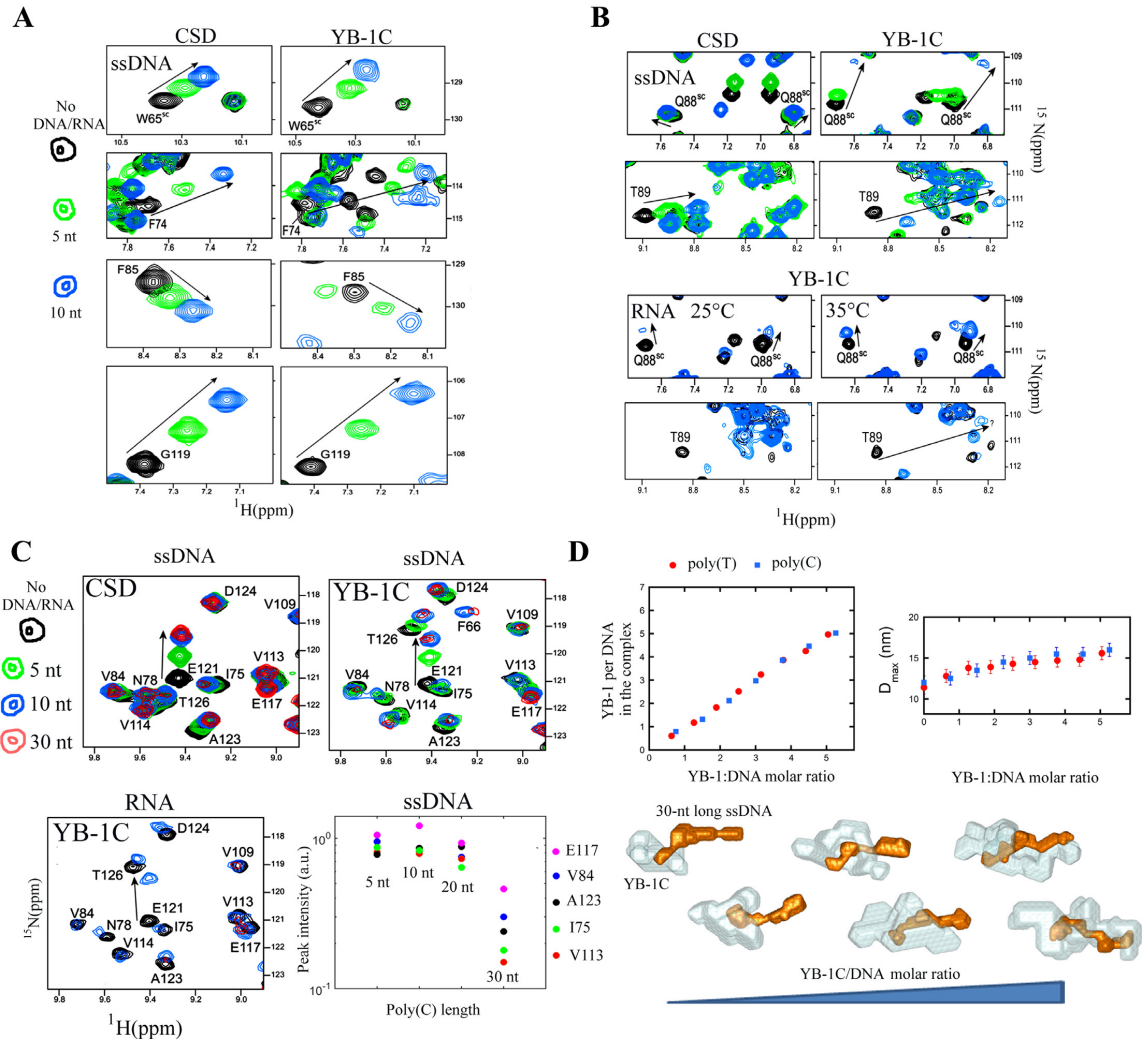
YB-1C binds to ssDNA/RNA through conserved CSD residues and forms an oligomer with 30 nt-long oligonucleotides. (**A**) Two-dimensional ^1^H-^15^N HSQC spectra of either YB-1C (aa 1–180) or CSD (aa 51–129) in the presence of poly(C) oligonucleotides (5 or 10 nt-long). View of conserved CSD residues that interact with DNA/RNA. (**B**) CSD residues that interact differently with nucleic acids in YB-1C compared to isolated CSD. (**C**) ^1^H-^15^N HSQC spectra of either YB-1C or CSD interacting with nucleic acids of different length as indicated. Lower right panel: heights of resonance peaks versus DNA oligonucleotide length (Poly(C)). (**D**) SAXS analysis of the binding of YB-1C to 30 nt-long Poly(C) or Poly(T) ssDNA. Upper left panel: number of YB-1C bound to ssDNA at different YB-1C concentrations. The number of ssDNA moles was kept constant and equal to 8.3 μM (Poly(C)) or 7.0 μM (Poly(T)). Upper right panel: maximal extension *D*_max_ of the scattering objects. *D*_max_ was deduced from the SAXS curves using the program GNOM. Poly(C) or Poly(T) DNA of 30 nt. Lower panel: model of YB-1C oligomerization on DNA provided by SAXS data for increasing values of the mixing ratio, R. The envelopes obtained using the program MONSA are shown with YB-1 depicted in light blue and 30 nt-long Poly(C) ssDNA in orange.

To probe whether the CSD structure and its interactions with RNA/ssDNA were affected by the presence of unstructured N- and C-terminus of YB-1C, we compared the structures of YB-1C (aa 1–180) and isolated CSD (aa 52–129, as studied in a previous report (43)) and their binding to oligonucleotides. NMR spectra revealed an overall similar structure for both isolated CSD and CSD within YB-1C, reflecting the proper folding of the *β*-barrel in YB-1C (Supplementary Figure S2a). Several conserved residues in CSD (44) (W65, F74, F85, G119) interacting with short RNA/ssDNA oligonucleotides (Poly(T), Poly(C) DNA/ Poly(U) and Poly(C) RNA) also show similar chemical shift variations in isolated CSD and YB-1C (Figure 3A and Supplementary Figure S3a). However, some discrepancies were observed since residues Q88 and T89 present in a short turn, T1 (aa, 88–92), located at the C-terminus of β3, experience larger chemical shift variations with ssDNA/RNA in YB-1C compared to isolated CSD (Figure 3B). The unstructured CTD probably reinforces the ability of T1 to interact with nucleic acids to secure the binding of 4 nt-long nucleic acids to the YB-1C CSD (Supplementary Figure S2b).

Having confirmed preserved interactions of the CSD of YB1C with short ssDNA/RNA (5 and 10 nt), we then explored whether longer oligonucleotides could generate YB-1C oligomerization that would be representative of the nucleoprotein filaments observed by AFM. Increasing the length of Poly(C) DNA from 10 to 20 nt leads to neither additional chemical shift variations nor significant peak broadening in CSD residues (Figure 3C). Therefore, all possible stacking and polar interactions have been engaged between YB-1C CSD and 10 nt-long oligonucleotides, and no dimerization or oligomerization is taking place in these conditions. Nevertheless, broadening of the peaks was detected for CSD residues when the length of Poly(C) DNA oligonucleotides was increased from 20 to 30 nt (Figure 3C). Similarly, increasing ssDNA/RNA oligonucleotide length from 10 to 30 nt, independently of nucleotide base (Poly(C), Poly(T), Poly(U)), generates significant broadening of amino acid peaks in NMR spectra (Supplementary Figure S2b and c). Peaks undergoing broadening are mostly localized in CSD indicating multiple bindings of YB-1C to 30 nt-long RNA/ssDNA (Supplementary Figure S3c). In an analogous manner, isolated CSD also displays peak broadening with 30-nt long DNA (Supplementary Figure S2b). The profile of peak broadening is also similar for isolated CSD and YB-1C CSD residues suggesting a similar spatial organization of CSDs contacting 30 nt-long DNA in YB-1C (Supplementary Figure S3d).

To obtain more information about the structure of 30 nt-long ssDNA/RNA complexed to YB-1C, we analyzed the structure of YB-1C:ssDNA complexes in solution by SAXS at increasing YB-1C/ssDNA molar ratios (Figure 3D and Supplementary Figure S4). We noticed a progressive increase in the YB-1C:ssDNA stiffness that may reflect the establishment of a rigid structure in which DNA and protein mobility are restrained (Figure 3D and Supplementary Figure S4). At the highest YB-1C/ssDNA molar ratio (5.05 for Poly(C)), ssDNA drives the formation of a complex of elongated shape with at least 5 YB-1C proteins per 30-nt long ssDNA, thus corresponding to 6 nt per YB-1C, consistently with the results of gel mobility shift assays (Figure 2C). Further increasing of YB-1C levels generates large aggregates probably because saturation has been reached. We then estimated the positions of YB-1C proteins on long ssDNA at different YB-1C/ssDNA ratios through the *ab initio* method (Figure 3D). Restoring the low resolution shapes of DNA:protein complexes at different YB-1C/ssDNA molar ratios reveals the binding of an isolated YB-1C to ssDNA ends at low YB-1C/ssDNA molar ratio. Then, upon increasing the YB-1C:ssDNA ratio, additional YB-1C proteins pile up one after another to cover most ssDNA nucleotides. The length of the elongated filament is about 16 nm for 30 nt (Figure 3D). Altogether, SAXS and NMR results are consistent with YB-1C oligomerization taking place on 30-nt long single stranded nucleic acids.

### Structure of the nucleoprotein filament

**Figure 4.**
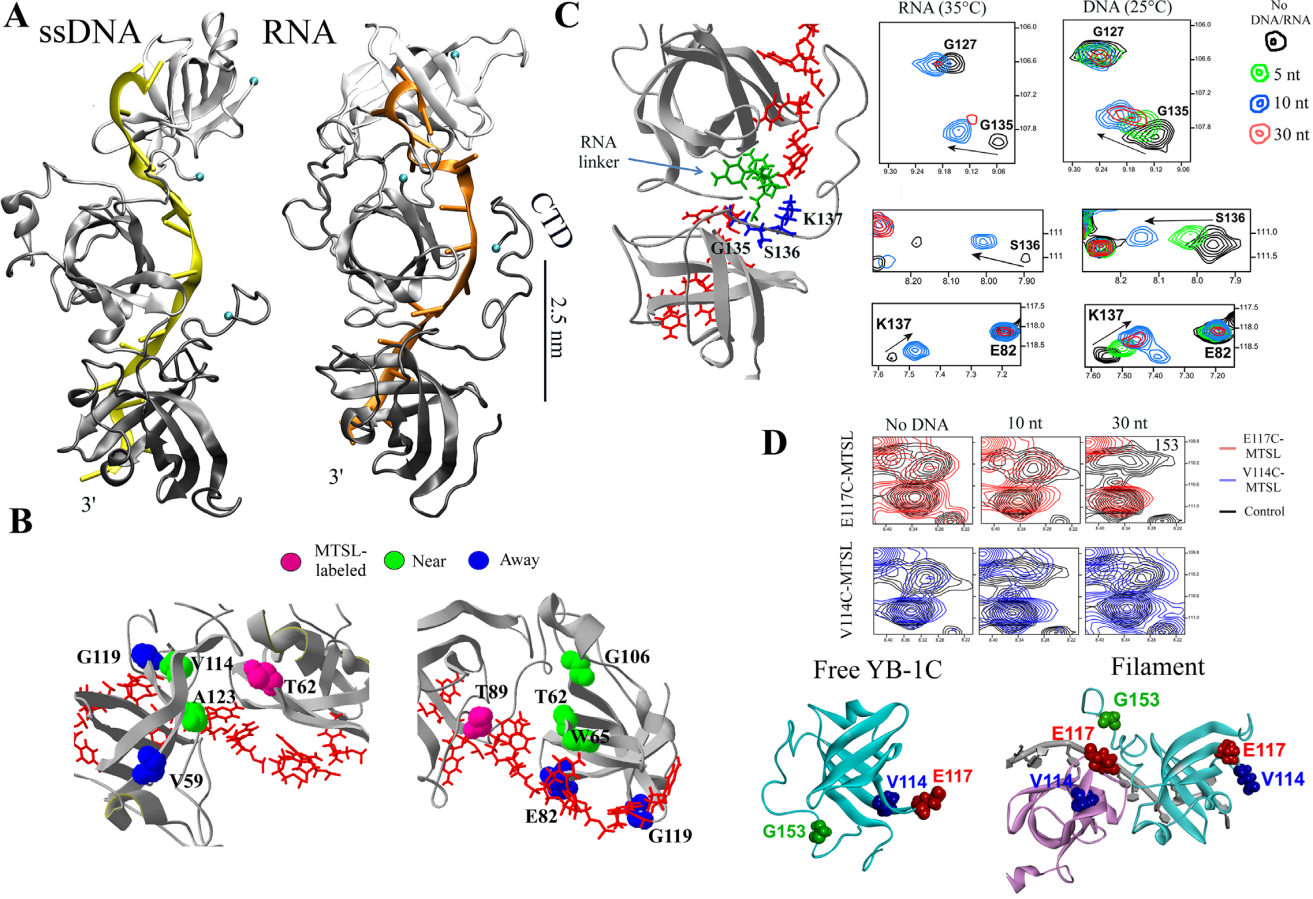
Structure of the nucleoprotein filament. (**A**) MD structures of ssDNA/RNA nucleoprotein filaments. Poly(C) DNA/RNA. 16 nt-long ssDNA/RNA. See ‘Materials and Methods’ section for details. Blue spheres represent protein C-terminus. (**B**) Relative positions between MTSL-labeled residues and nearby or away residues (according PRE data) reported in the MD structure. (**C**) Two-dimensional ^1^H-^15^N HSQC spectra of either YB-1C (aa 1–180) or CSD (aa 51–129) showing G135, S136 and K137 residues in the presence of RNA/ssDNA oligonucleotides as indicated. (**D**) According to PRE data (using E117C and V114C-MTSL mutants), G153, a residue located in the CTD of YB-1C, is relocated near the E117 residue of an adjacent CSD in the presence of 30-nt long DNA.

MD was used to propose a model of the nucleoprotein filament structure and to reveal interactions leading to its stability. The starting configuration consists of three YB-1C interacting with a 16 nt-long Poly(C) ssDNA/RNA oligonucleotide to mimic a short nucleoprotein filament fragment (see ‘Materials and Methods’ section for details). Four consecutive ssDNA/RNA nucleotides per one CSD interact with aromatic residues located in the *β*-barrel of YB-1C (Figure 3A and B; Supplementary Figure S3a). Then, a linker of two additional nucleotides was added to join adjacent CSDs leading to 6 nt per YB-1C, in agreement with SAXS results (Figure 3D). The CTD of the last YB-1C protein had to be removed to respect the symmetry and prevent boundary problems that impair the stability of the complex. MD then shows the formation of a quite stable complex with 16 nt-long RNA or ssDNA (Figure 4A, Supplementary Figure S5 and video 1). Single-stranded RNA or DNA is channeled between YB-1C proteins which are tilted relative to each other in order to allow the passage of nucleic acids. Thus, DNA/RNA is not compacted but rather follows a helical path by channeling between YB-1C proteins. The separation distance between consecutive CSDs is about 2.5 nm. Given the maximum extension, of the 30 nt-long ssDNA/YB-1C filaments (∼16 nm) measured by SAXS (Figure 3D), the filament length of the MD model (∼8 nm for 16 nt) is a good match.

To explore experimentally the spatial organization of YB-1C in the nucleoprotein filament structure, 5 YB-1C MTSL-labeled mutants (T62C, T89C, T108C, V114C, E117C) were prepared and mixed with either 10 or 30 nt-long ssDNA to which YB-1C binds as a monomer or forms oligomers, respectively (Figure 4B; Supplementary Figures S6 and 7). PRE then provided proximity information between the MTSL-labeled residue (cysteine) and unlabeled residues in the nucleoprotein filaments. This analysis was restricted to 30-nt ssDNA since resonance peaks were detected with a higher signal to noise ratio. Indeed, with 30-nt RNA, we observed a larger peak broadening that may reflect a more rigid structure. We confirmed that there was only one YB-1C interacting with a 10 nt-long Poly(C) DNA since only intramolecular PREs were observed (Supplementary Figure S6b). Intermolecular PREs in the presence of 30 nt-long Poly(C) DNA then revealed couples of residues in close proximity (T62C with V114 and A123; T89C with W65, T62 and G106; T108C with F111, V114, A123 and T124; V144C with T62, G106 and G129; E117C with T62, T108, V109 and F111) in good agreement with the filament structure predicted by MD (Figure 4B and Supplementary Figure S7).

In addition, we identified residues experiencing the most dramatic peak broadening after the formation of the nucleoprotein filaments for both DNA and RNA oligonucleotides. Consistent with reduced exchange dynamics and(or) reduced mobility due to steric hindrance, many of these residues are located at the interface between two consecutive CSDs in the MD structure (Supplementary Figure S8a and b).

In summary, both NMR and MD point toward the formation of a nucleoprotein filament in which ssDNA/RNA are channeled in between closely spaced CSDs.

### The deployment of the basic C-tail onto adjacent CSD leads to stabilization of the nucleoprotein filament and electrostatic neutralization of RNA/ssDNA

Forming a nucleoprotein filament most probably involves weak forces that are not sufficient to secure the formation of YB-1C oligomers on nucleic acids shorter than 20 nt under NMR experimental conditions (Figure 3C). Accordingly, chemical shift variations of CSD residues upon increasing the length of ssDNA or RNA from 10 to 30 nt reveals no strong additional interactions, including among conserved residues interacting with nucleic acids (W65, F74, F85, G119) (Supplementary Figure S6a). Moreover, CSD alone could not form a nucleoprotein filament (Figure 1B). We then considered the roles of the N- and C-terminal unstructured domains flanking the CSD in YB-1C. As residues located in the N-terminal do not show significant variations in the peak heights in the presence of 30-nt long DNA/RNA (Supplementary Figure S8a), we restricted our study to the CTD of YB-1.

**Figure 5.**
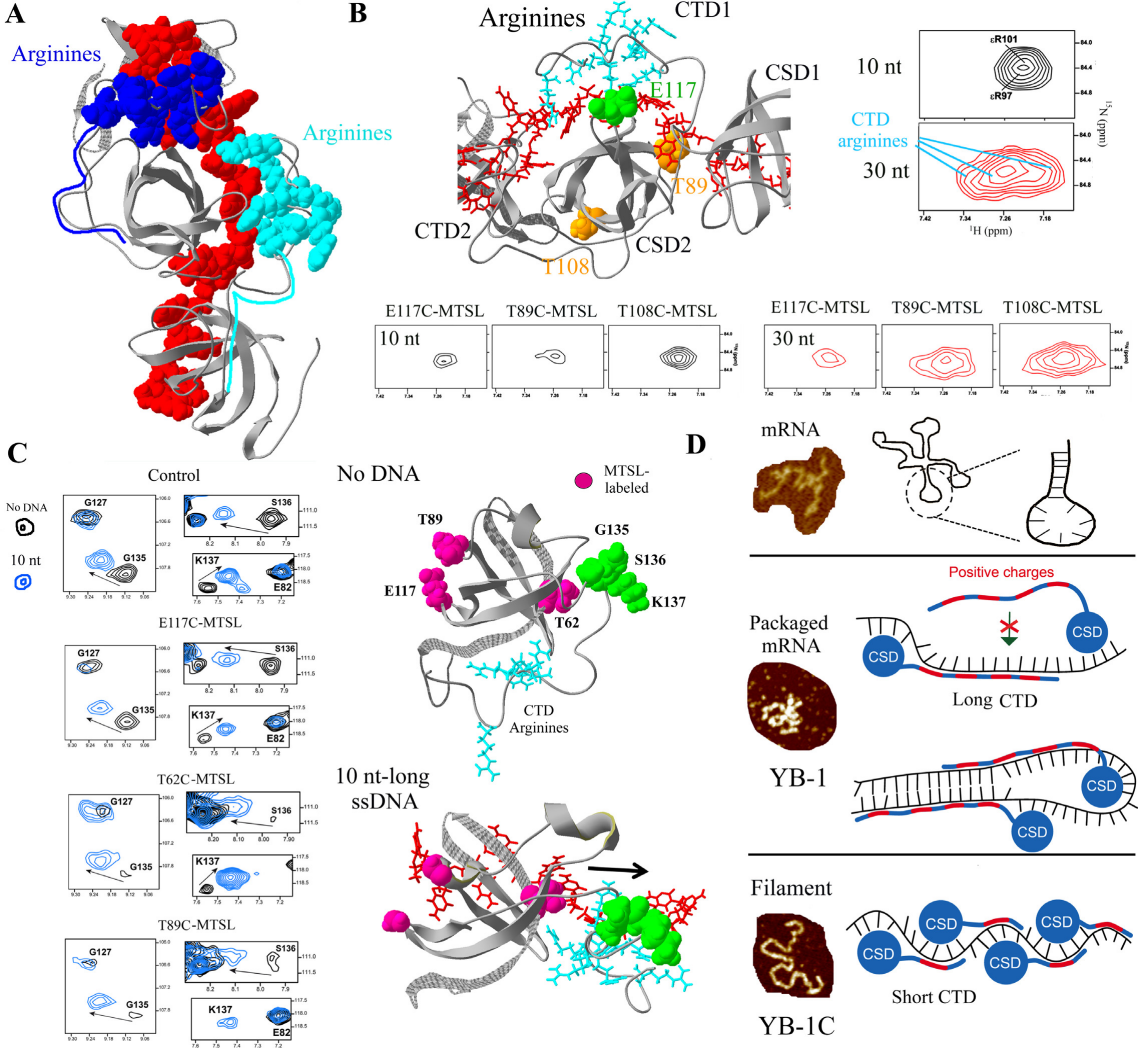
YB-1 forms a nucleoprotein filament along ssDNA/RNA through an electrostatic zipper mechanism. (**A**) View of the location of CTD arginine residues (blue) and RNA in the filament (red). MD structure of RNA nucleoprotein filaments. (**B**) Resonance peaks of arginine side chains with or without spin-labeled residues as indicated. Upper left panel: relative position of CTD arginine residues and spin-labeled residues in the RNA filament structure (MD structure). Poly(C) DNA. (**C**) Deployment of CTD residues G135, S136 and K137 after the binding of YB-1C to a 10-nt DNA, as revealed by PRE experiments. Left panel: PRE data. Upper panel: view of positions of spin-labeled residues (T62C, T89C and E117C), CTD arginine residues, G135, S136 and K137 in the presence or absence of 10 nt-long DNA (MD structure with DNA). The arrow indicates the putative movement. (**D**) Model of structural transition from packaged mRNPs to linear mRNA nucleoprotein filaments. The short CTD of YB-1C enables the formation of a nucleoprotein filament via an electrostatic zipper mechanism which disrupts mRNA secondary structure. The long CTD of YB-1 prevents long-ranged oligomerization along mRNA and preserves mRNA secondary structures. Multiple phosphorylation events in CTD residues, interactions with protein partners and electrostatic interactions with positively charged CTD residues may trigger such structural transition in cells.

We noticed that the YB-1C CTD extends toward an adjacent YB-1C in the nucleoprotein filament (Figure 4A). The unstructured and basic CTD may thus bridge adjacent CSDs to promote the oligomerization process. In agreement with predicted CTD deployment, CTD residues such as G153 are located in the vicinity of E117C, in a nearby CSD in the filament (Figure 4D). In addition, when increasing ssDNA/RNA length from 10 to 30 nt, we observed a significant peak broadening of some CTD residues between aa 130–156, such as N143 and Y145, but not for residues 166–180, located away from the CTD arginine-rich cluster (Supplementary Figure S8a and d). Interestingly, molecular modeling shows the interaction of arginine residues (R142, R146–147, R150–153, R156) with RNA/ssDNA phosphates at the surface of a nearby CSD (Figure 5A). In NMR spectra, arginine residues located in the CTD were only detected at 25°C and in the presence of 30 nt-long ssDNA (Figure 5B and Supplementary Figure S9). In the absence of ssDNA, arginine side chains of unstructured CTD residues cannot be detected, most probably because of rapid exchange with water. The interaction of arginine residues with ssDNA phosphates reduces solvent-exchange dynamics, leading to their appearance at this characteristic chemical shift ((61), Supplementary Figure S9). We took advantage of their detection to probe their location in the ssDNA nucleoprotein filament. PRE data confirm the presence of the CTD arginine residues in the vicinity of nucleotides interacting with a nearby CSD only when oligomerization takes place (spatial proximity with E117, Figure 5B). Although the location of the unstructured CTDs shows a large variability in the MD model of RNA/ssDNA nucleoprotein filaments, some CTD residues (G135, S136 and K137) appear to be located near the RNA linker, between two consecutive CSDs, presumably to orient arginine CTD toward a nearby YB-1C CSD (Figure 5c). The analysis of NMR data indeed revealed significant chemical shifts occurring for G135, S136 and K137 residues after the binding of YB-1C to either ssDNA or RNA (Figure 4C). The amplitude and direction of the chemical shifts also depend on the nature and the length of nucleic acids (Figure 4C and Supplementary Figure S8c) as expected in the presence of neighboring nucleic acids. To probe the CTD movements, we also noticed that G135, S136 and K137 residues are initially close to their own CSD protomer in the absence of ssDNA. However, after the binding of YB-1C to 10 nt-long ssDNA, these residues are displaced away from their own CSD (Figure 5C), suggesting a long-range CTD movement. The affinity of CTD arginine residues for the accessible RNA/ssDNA sugar–phosphate backbone protruding away from a nearby CSD may simply by itself lead to the CTD deployment in the nucleoprotein filament.

In the nucleoprotein filament, each YB-1C CTD enables the neutralization of sugar-phosphate backbone charges corresponding to ssDNA/RNA nucleotides that interact with CSD aromatic residues of an adjacent YB-1C. Secondary mRNA/ssDNA structures, even with short stems, would prevent YB-1C oligomerization since CSD has little affinity for dsRNA and then could no longer be tightly packed along RNA. We propose that the energy benefit resulting from an electrostatic zipper promotes a chaperone activity that disrupts folded nucleic acid secondary structures to allow the formation of the linear filament (Figure 5D). In the CTD of full length YB-1, there are three additional clusters of positive charges (Figure 1A) that might neutralize many nucleotides around the CSD to refrain long-ranged oligomerization along mRNA/sDNA. In line with this hypothesis, YB-1 binds to about 20–30 nt (56), which is much longer than the 6 nt measured by SAXS with YB1-C (Figure 3D). In addition, this model is consistent with preserved mRNA secondary structure observed in the presence of YB-1, in contrast to YB-1C (Figure 2C). However, under certain conditions, multivalent CTD–CTD interactions due to the presence of alternating regions of predominantly basic or acidic amino acids (Supplementary Figure S10c), phosphorylation events in the CTD (34,35), or interaction with protein partners, may partly displace the CTD from mRNA and therefore allow the formation of a nucleoprotein filament with mRNA.

## DISCUSSION

mRNA translation depends on many factors including RNA helicases, translation initiation factors (62–64) that bind to 5′ UTRs and 3′ UTRs, whose activities are regulated by post-translational modifications (65) and modifications in mRNA itself (8). When the 43S pre-initiation complex (43S PIC) encounters mRNA, it recognizes the cap structure (m7GpppN) and starts scanning mRNA until it reaches and identifies the initiation codon in a suitable sequence context (66). After the GTP hydrolysis, dissociation of eIF2 and recruitment of 60S subunit, 80S ribosome proceeds through mRNA coding sequences for synthetizing polypeptide chain and is able itself to disrupt mRNA secondary structures (67). The 5′ UTRs of mRNAs could also possess strong secondary structures that can affect scanning of mRNA by 43S PIC. The helicases, such as eIF4A, have been particularly studied owing to their capacity to unwind structured mRNA regions enabling the recognition and progression of the 43S PIC through the highly structured 5′ UTRs (68,69). While helicases are able to disrupt secondary structures of mRNA that block translation, likely, after their passage, mRNA would become compacted due to the flexible nature of free RNA (70) or mRNA secondary structures would reform. Protein factors that could stretch and stabilize mRNA in a linear conformation, after the unwinding by helicases, are probably required to increase the accessibility/processivity of 43S PIC and ribosomes. An analogy can be made with SSBs that help to handle single stranded DNA for replication and recombination, along with DNA helicases. Indeed, both in bacteria and eukaryotes, SSB Proteins (36), RAD51 (71), gp32 (72) and RecA (73) polymerize along ssDNA to form a nucleoprotein filament. In this regard, it is tempting to speculate that similar proteins may exist to facilitate the scanning of mRNA by large molecular complexes such as the 43S PIC and ribosomes.

Here we show, that YB-1C, a truncated form of YB-1, has the capacity to form linear nucleoprotein filaments with mRNA through the conserved RNA chaperone activity of the CSD (Figures 1C and 2A). This, to our knowledge, is the first mRNA-binding protein capable to stabilize RNA in a linear conformation. The formation of a nucleoprotein filament relies on an electrostatic zipper mechanism, where mRNA complexed with YB-1C is channeled in between consecutive CSDs (Figure 4D). The basic YB-1C CTD is deployed and bridges nearby CSD, thereby neutralizing RNA phosphates exposed toward its arginine residues. In eukaryotes, a cluster of basic amino acid residues in CTD, just after the CSD, is always present despite its quite limited sequence conservation (Supplementary Figure S10a). Interestingly, residues located in between CSD and the first basic cluster of CTD show a significant conservation, suggesting a potential role in orienting CTD basic residues toward adjacent CSDs. In contrast, the N-terminus of YB-1 does not show any sequence conservation. In bacteria and plants, CSPs have no positively charged cluster near CSD, what could explain why the elevated levels of CSP are required to unwind secondary structures of mRNAs (33).

While the advantage of forming a linear nucleoprotein filament to facilitate the scanning by large machineries involved in mRNA translation is evident, one may wonder whether such nucleoprotein filament can be formed in a cellular context. Full length YB-1 packages mRNA to inhibit translation *in vitro*, most probably due to the presence of additional clusters of basic residues in the CTD. Binding of protein partners or post-translational modifications in the CTD, which harbors many phosphorylation sites (34,35) (Supplementary Figure S10b), may neutralize the strong positive charge of CTD and trigger the formation of a linear nucleoprotein filament in cells, in the same way as YB-1C. Consistent with a role of YB-1 phosphorylation in mRNA activation, Akt kinase restores translation of silent mRNA by phosphorylating YB-1, though the release of YB-1 from mRNA was then proposed to explain mRNA activation (74). Alternatively, CTD could be engaged in multivalent interactions with itself due to its alternating positively and negatively charged clusters (Supplementary Figure S10c) to neutralize the RNA packaging activity of the CTD. At YB-1:mRNA ratios above the saturation, displacement of a part of the CTD, that prevents the formation of a nucleoprotein filament (aa 198–324), has already been detected *in vitro* (56). In addition, in cells, nucleoprotein filaments could be formed transiently, in concert with RNA helicases, and occur on short mRNA segments rather than along the entire mRNA length. Due to the abundance of YB-1 in translationally silenced mRNPs (14–17), the structural plasticity of YB-1:mRNA complexes may play a key role in the activation of translation of these mRNAs.

Recent crosslinking and immunoprecipitation (CLIP) data obtained for YB-1 in glioblastoma cells provided clues about the way YB-1 interacts with mRNA *in vivo* (75). YB-1 binds to coding sequences (36%) and to lesser extent to the 5′ UTR (< 5%) but it has a preference for 3′ UTR (51%). Considering the binding of YB-1 to active mRNA and not only to inactivated mRNA, we might expect that 5′ UTR and coding sequences will be stripped of YB-1 by the scanning 43S PIC and ribosomes, resulting in an apparent preference of YB-1 for 3′ UTRs. In addition, the continuous footprint left by YB-1 in certain mRNAs including coding sequences is consistent with the formation of a nucleoprotein filament (Supplementary Figure S10c). However, CLIP data represent an average YB-1 binding over many different mRNA states. CLIP footprints for YB-1, even compatible with the formation of a nucleoprotein filament, cannot be used as the evidence for its existence. Future investigations through cellular approaches are needed to reveal the putative presence of mRNA filaments in mammalian cells and, the putative roles of YB-1 phosphorylation and of its protein partners in translation regulation in cells. As YB-1 protein levels are frequently elevated in different types of human cancers (76), the formation of linear mRNA nucleoprotein filaments may provide a basis for enabling elevated rates of protein synthesis taking place in cancer cells.

The ability of YB-1C to form a nucleoprotein filament with ssDNA also raises the possibility that YB-1 may play an important role in ssDNA processing by large molecular machineries in the nucleus. The cleavage of YB-1, notably by the 20S proteasome after residue G219 upon genotoxic stress (77), directs the protein to the nucleus, possibly to exert functions related to alternative slicing (78) and ssDNA processing. Notably, when in the nucleus, YB-1 may enable the formation of ssDNA nucleoprotein filaments, participate in DNA repair (79) and contribute to cancer drug resistance (80).

## Supplementary Material

Supplementary Data
