# Supplementary material for: YB-1, an abundant core mRNA-binding protein, has the capacity to form an RNA nucleoprotein filament: a structural analysis
Source: Nucleic Acids Res. 2019 Jan 3;47(6):3127–41. doi: 10.1093/nar/gky1303 (PMC6451097; doi:10.1093/nar/gky1303)
Supplement: Supplementary Data [file gky1303_supplemental_files.zip › Legends and Supplementary Figures 1 to 10.pdf]

## Supplementary figure legends:

Figure S1: *In vitro* translation assay shows that YB-1C does not inhibit protein synthesis.
**a**, Red Ponceau staining used to confirm the concentration of YB-1C and YB-1. The anti-YB-1 antibody used in Fig 2d better recognizes FL YB-1 than YB-1C in the presence of rabbit reticulocyte lysate, which may leave an impression that YB-1C was used at lower concentration than YB-1 in translation assays. We then performed several controls to estimate the relative concentration of YB-1 and YB-1C.
**b**, *In vitro* translation of mRNA Luciferase (4 nM) pre-incubated in the presence of indicated concentrations of YB-1C (see Materials and Methods for details).
**c,** Upper panel: *In vitro* translation of mRNA Luciferase (4 nM) either pre-incubated in the presence of indicated concentrations of YB-1C or added to rabbit reticulocyte lysates 30 s after YB-1C. Note the higher rate of translation of the preformed YB-1C:mRNA filaments (their formation was controlled by AFM).

Figure S2: NMR spectra of the YB-1 constructs used in this study.
**a**, $^{1}$H-$^{15}$N HSQC spectra of truncated YB-1 mutants. The residues located in the CSD structure are not visible in the longer construct (aa, 1-219). A similar pattern is observed for full length YB-1 (data not shown). T = 25°C.
**b**, $^{1}$H-$^{15}$N HSQC spectra of YB-1C or isolated CSD in the presence of Poly(C) ssDNA of increasing length at 25°C.
**c**, $^{1}$H-$^{15}$N HSQC spectra of YB-1C in the presence of RNA oligonucleotides and of increasing length at 25°C. We noted the chemical shifts of conserved residues known to interact with nucleic acids.

Figure S3: Putative structural differences between isolated CSD and YB-1C CSD.
**a**, $^{1}$H/$^{15}$N combined chemical shift variations of CSD and YB-1C residues when interacting with 10nt-long Poly(C) ssDNA at 25°C.
**b**, Left panel: $^{15}$N-$^{1}$H NMR spectra of YB-1C or CSD at 25°C. Residues Q88, T89, T126 and G127 do not display similar chemical shifts in the two CSDs.
Right Panel: View of the location of the residues in the CSD structure(1). The four residues are located near CSD C-terminus.
**c**, Peak height ratio of YB-1C residues interacting with 30 and 10 nt-long ssDNA (Poly(C)). Note the dramatic reduction in peak height of residues located in CSD and in a part of the CTD.
**d**, Peak height variations of CSD residues when either YB-1C or isolated CSD interacts with 30 nt-long Poly(C) DNA.

Figure S4: SAXS analysis of YB1-C:DNA complex.
**a**, Scattering curves I(q) for each value of the YB-1C/oligonucleotide molar ratio, R, in two cases, Poly(C) and Poly(T).
**b**, The same curves shown using a dimensionless Kratky representation, $(qR_g)^2I(q)/I(0)$ as a function of $qR_g$. These Kratky plots are indicative of the evolution from a fully unstructured object (gray curve, ssDNA alone) towards a more compact and rigid object (red curve).

Figure S5: Molecular dynamics of the mRNA nucleoprotein filament.RMSD of backbone residues (red, protein; black, RNA) versus time for the last 100 ns of the 300 ns production phase. The RMSD variations do not exceed 0.5 nm and represent stable state for each system.

Figure S6: YB-1C oligomerization evidenced by PRE.
**a**, Histograms of peak heights for spin-labeled YB-1C. NMR spectra of MTSL-labeled $^{15}$N-YB-1C in the presence of 10 or 30 nt-long Poly(C) DNA. While interacting with 10 nt-long DNA, only residues located near MTSL-labeled residues in the CSD structure display a significant decrease in peak heights compared to unlabeled YB-1C. However, in the presence of 30 nt-long DNA, additional residues display a marked decrease in peak height. As the structure of YB1 CSD is preserved, YB-1 oligomerization along DNA can explain this pattern which was further demonstrated by PRE data.
**b**, Chemical shift variation of YB-1C residues at 25°C when interacting with 10 and then 30 nt-long Poly(C) ssDNA. No large chemical shift variations were detected with 30 nt compared to 10 nt.

Figure S7: PRE analysis of the YB-1C:YB-1C interface in the filament.
Five YB-1C mutants with single spin-labeled residues (MTSL) were produced after their mutation to cysteine (No cysteine residue is present in YB-1C). We estimated the proximity between residues in the nucleoprotein filament by increasing the length of the Poly(C) DNA nucleotide from 10 to 30 nt. YB-1C oligomerization only takes place in the presence of 30 nt-long DNA.
NMR spectra are shown for either MTSL-labeled YB-1C mixed with $^{15}$N-YB-1C (1:1 ratio, T62C and V114C) or MTSL-labeled $^{15}$N-YB-1C alone (T62C, V114C, T108, E117C, T89C), as indicated. Spectra of $^{15}$N-YB-1C were used as controls to show peak broadenings resulting from oligomerization on 30-nt long DNA. Whenever MTSL-labeled residues are located near a $^{15}$N-labeled residue (< 20 Å), the heights of the corresponding resonance peaks should decrease. **a**, T62C, **b**, V114C, **c**, T108C, **d**, E117C, **e**, T89C. T= 25°C.

Figure S8: NMR analysis of peak broadening and chemical shifts occurring in CTD residues.
**a,** Histograms of the ratio of peak heights for YB-1C interacting with either 10 or 30 nt-long Poly(C) oligonucleotides. Note the sharp decrease in peak height of residue located in CSD for both ssDNA and RNA but also of residues located near the arginine-rich residues in the CTD. Only data from peaks of assigned residue backbones are shown.
**b**, View of the residues displaying the more dramatic peak broadening for both DNA and RNA in the RNA nucleoprotein filament (below the line in **a**).
**c**, $^{15}$N-$^{1}$H NMR spectra of YB-1C interacting with Poly(T) DNA or Poly(U) RNA oligonucleotides, as indicated. We noticed that chemical shifts were again present but different and of a lower amplitude than in the presence of Poly(C) DNA or RNA oligonucleotides (figure 3C).
**d**, Data showing the peak broadening of N143 and Y145 in the CTD in presence of 30 nt-long Poly(C) DNA. View on their putative location in the DNA nucleoprotein filament.

Figure S9: Detection in $^{1}$H-$^{15}$N HSQC spectra of CSD and YB-1C arginine guanidino Nε-Hε resonances in the absence or presence of poly(C) DNA (10 and 30 nt). CSD and CTD Arg Nε-Hε are labeled. In YB-1C, there are 11 arginine residues. Four are located in CSD and were assigned in the isolated CSD. Two of them are in the long unstructured loop in between β3 and β4 strands (R101 and R97). When YB-1C is complexed to 30 nt-long ssDNA, we noticed the appearance of additional peaks which have been

attributed to CTD arginine residues. Note that CTD arginine residues are not detected in the presence of 10-nt long ssDNA/RNA and 30-nt long RNA most probably for different exchange dynamics with the solvent. In general, interaction of YB-1C with RNA leads to a more dramatic peak broadening than with ssDNA.

Figure S10: Conserved residues in the CTD, CLIP data on YB-1 and putative phosphorylation sites.
**a**, Conservation of the C-or N-terminus sequences flanking CSD in eukaryotes, plants and bacteria.
Note the conservation of a cluster of basic residues in the C-terminus of YB-1 in eukaryotes which is missing in plants and bacteria (in blue). In addition, residues in between CTD basic residues and CSD are also partially conserved in eukaryotes (in green). CSD conserved residues (in yellow). The N-terminal domain of YB-1 is largely non-conserved and shortens progressively from mammals to bacteria.
**b**, Plot of the integrated charge density in a 5 amino acid window along YB-1 CTD reveals the presence of the 4 positively charged clusters in CTD. Most of the possible phosphorylation sites are located in between aa 140-210. We speculate that multiple phosphorylation events may interfere with the capacity of YB-1 to form a nucleoprotein filament.
**c**, CLIP data of YB-1 on EIF4G2 mRNA(2). EIF4G2 is considered here as a representative mRNA targeted by YB-1, when the number of counts registered were sufficient to account for a putative oligomerization. The YB-1 footprint along mRNA is so dense that it may be compatible with the formation of a nucleoprotein filament in cells. However this is not a proof by itself. We also noticed the decreasing CLIP counts from 5’UTR to 3’UTR. Igvtools (Integrative Genomics Viewer) was used to plot YB-1 footprints.

## REFERENCES:


1. Kloks, C.P., Spronk, C.A., Lasonder, E., Hoffmann, A., Vuister, G.W., Grzesiek, S. and Hilbers, C.W. (2002) The solution structure and DNA-binding properties of the cold-shock domain of the human Y-box protein YB-1. *Journal of molecular biology*, **316**, 317-326.
2. Wu, S.-L., Fu, X., Huang, J., Jia, T.-T., Zong, F.-Y., Mu, S.-R., Zhu, H., Yan, Y., Qiu, S. and Wu, Q. (2015) Genome-wide analysis of YB-1-RNA interactions reveals a novel role of YB-1 in miRNA processing in glioblastoma multiforme. *Nucleic acids research*, **43**, 8516-8528.

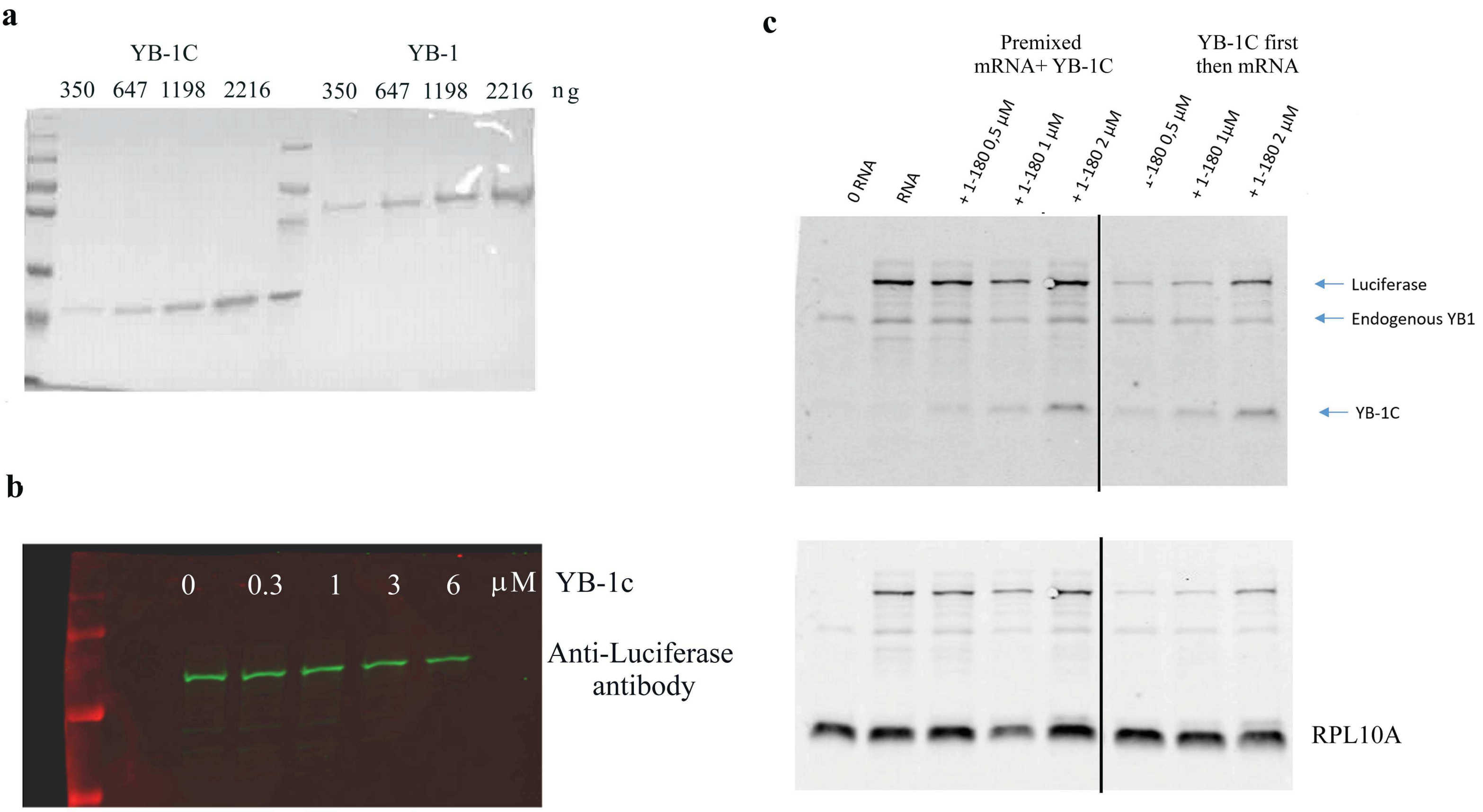

a
YB-1C
YB-1
350 647 1198 2216
350 647 1198 2216 ng
b
0 0.3 1 3 6 µM YB-1c
Anti-Luciferase antibody
c
Premixed mRNA+ YB-1C
YB-1C first then mRNA
0 RNA
RNA
+ 1-180 0,5 µM
+ 1-180 1 µM
+ 1-180 2 µM
+-180 0,5 µM
+ 1-180 1µM
+ 1-180 2 µM
Luciferase
Endogenous YB1
YB-1C
RPL10A


Figure S1

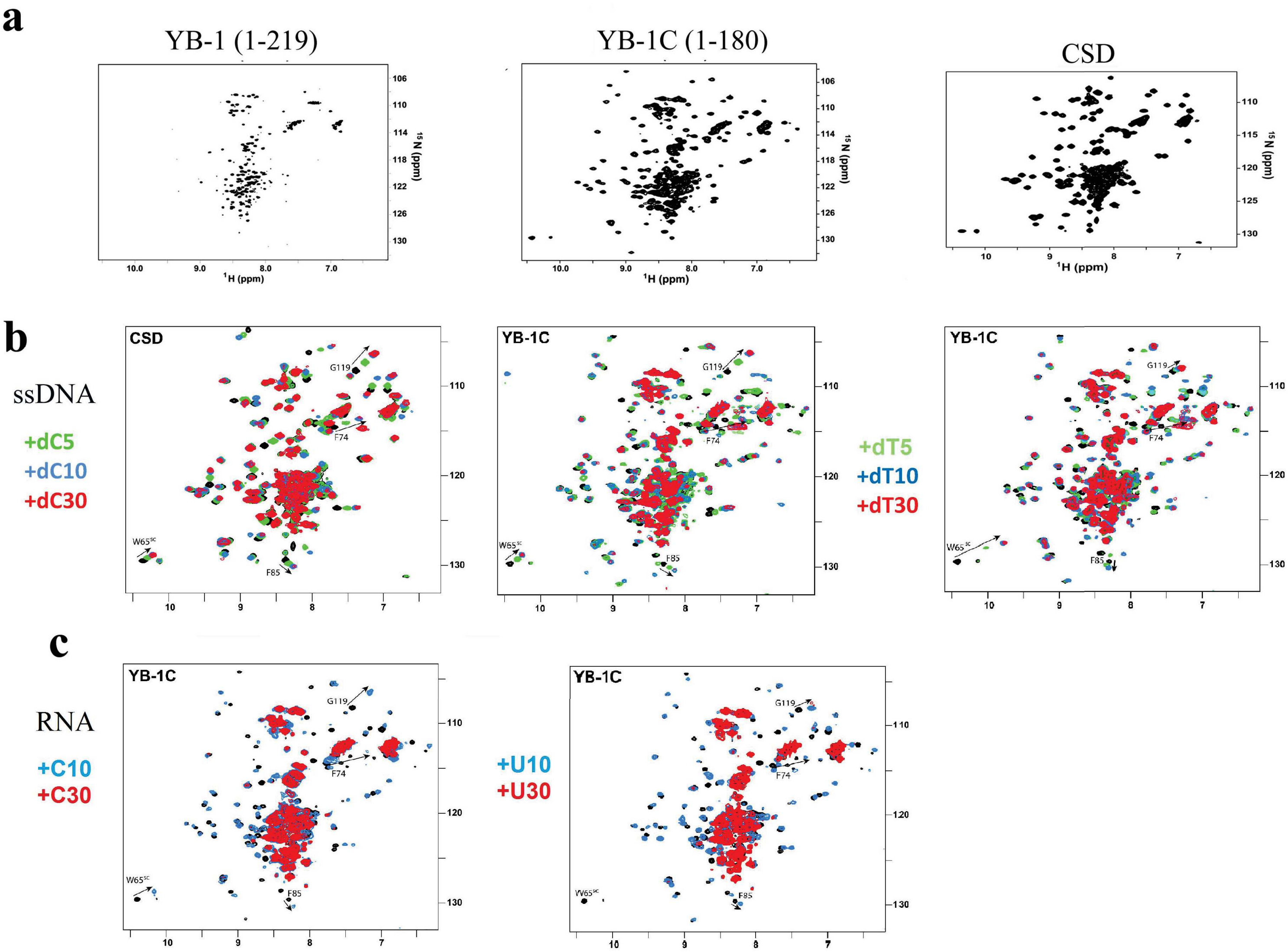
a
YB-1 (1-219)
YB-1C (1-180)
CSD
1H (ppm)
15N (ppm)
b
ssDNA
+dC5
+dC10
+dC30
CSD
YB-1C
+dT5
+dT10
+dT30
G119
F74
W65sc
F85
c
RNA
+C10
+C30
+U10
+U30

Figure S2

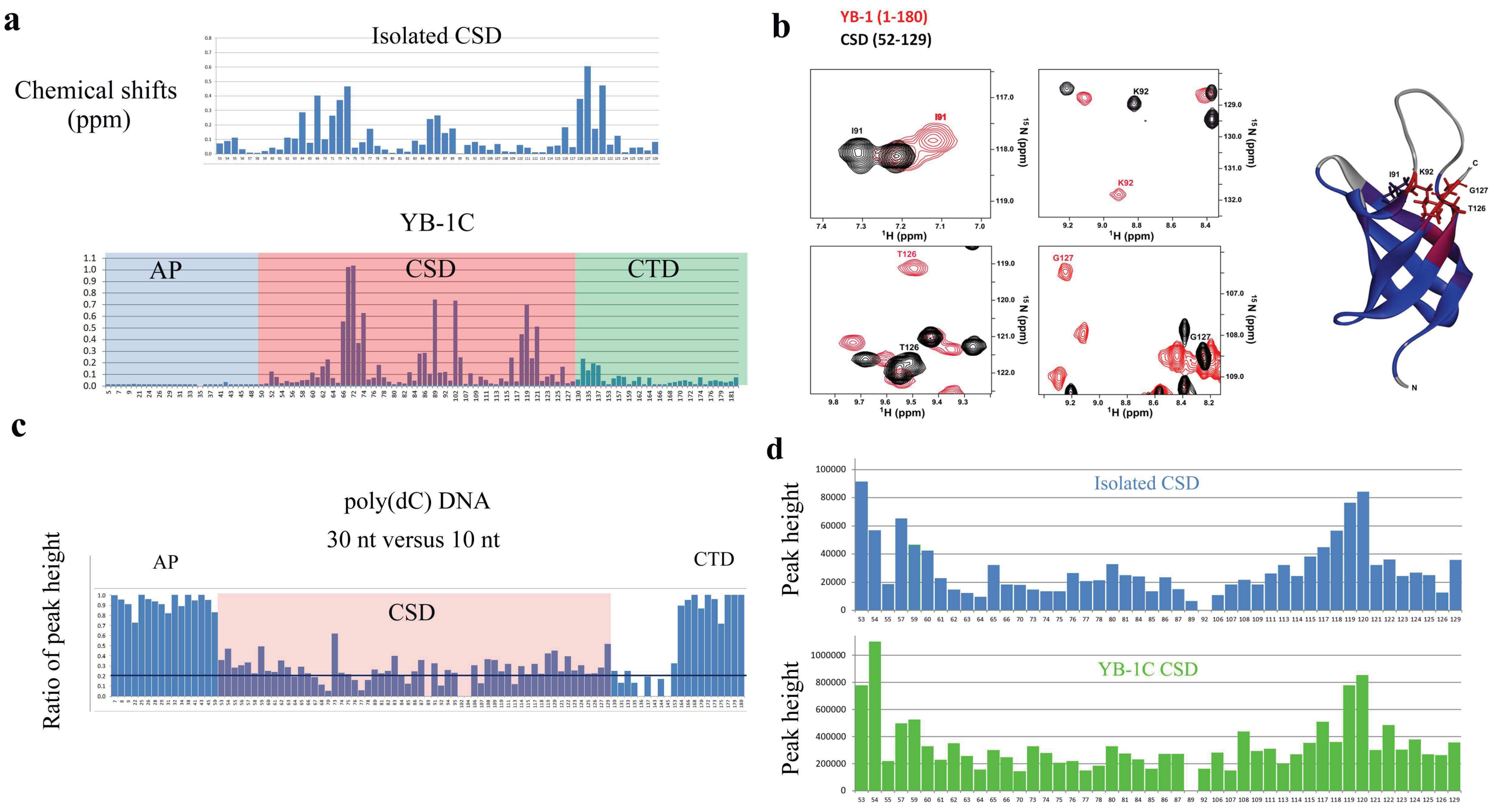
a
Chemical shifts (ppm)
Isolated CSD
YB-1C
AP
CSD
CTD
b
YB-1 (1-180)
CSD (52-129)
I91
K92
T126
G127
$^1$H (ppm)
$^{15}$N (ppm)
C
N
c
Ratio of peak height
poly(dC) DNA
30 nt versus 10 nt
AP
CSD
CTD
d
Peak height
Isolated CSD
YB-1C CSD

Figure S3

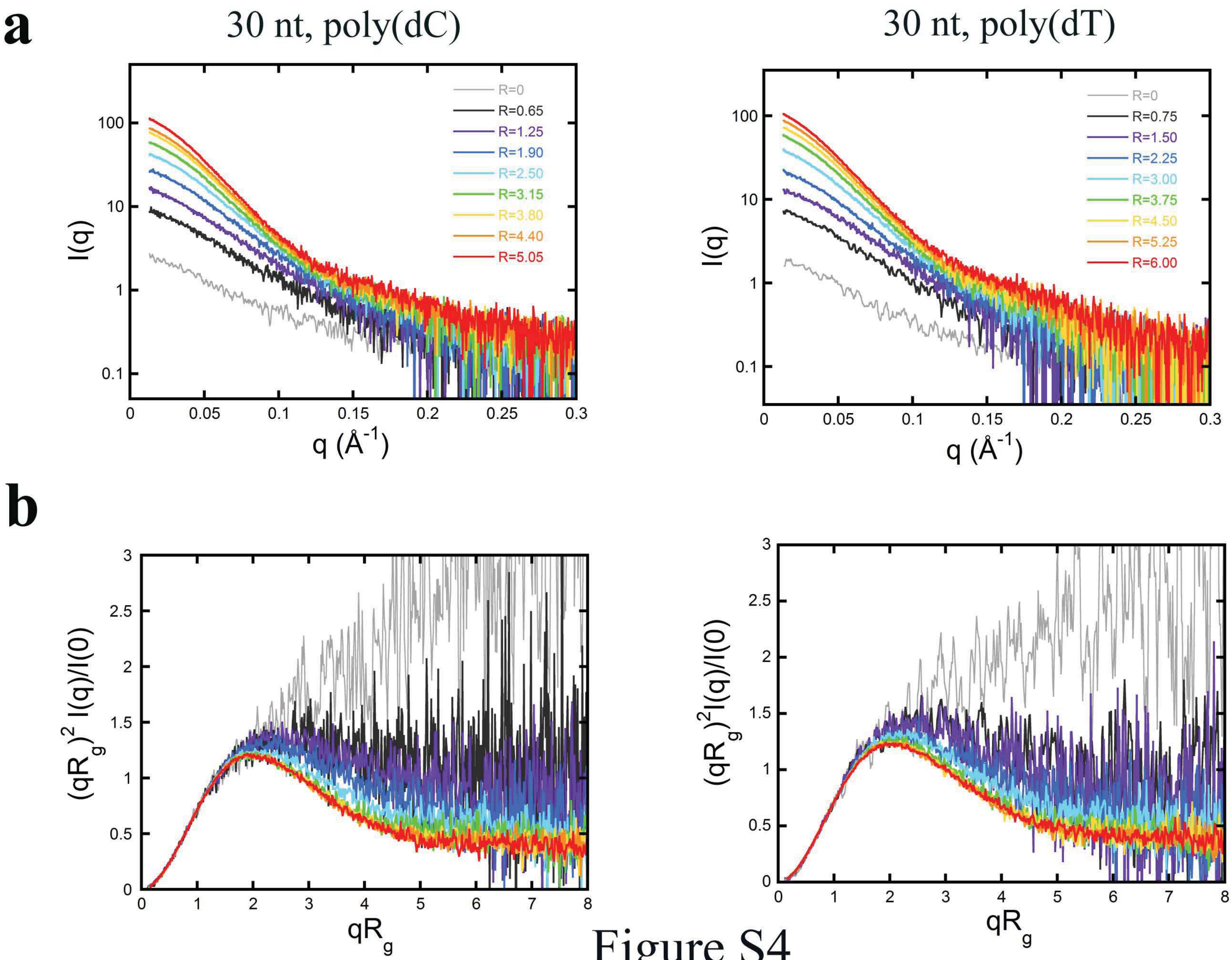

a
30 nt, poly(dC)
30 nt, poly(dT)
R=0
R=0.65
R=1.25
R=1.90
R=2.50
R=3.15
R=3.80
R=4.40
R=5.05
R=0.75
R=1.50
R=2.25
R=3.00
R=3.75
R=4.50
R=5.25
R=6.00
I(q)
q (Å$^{-1}$)
b
$(qR_g)^2$ I(q)/I(0)
$qR_g$


Figure S4

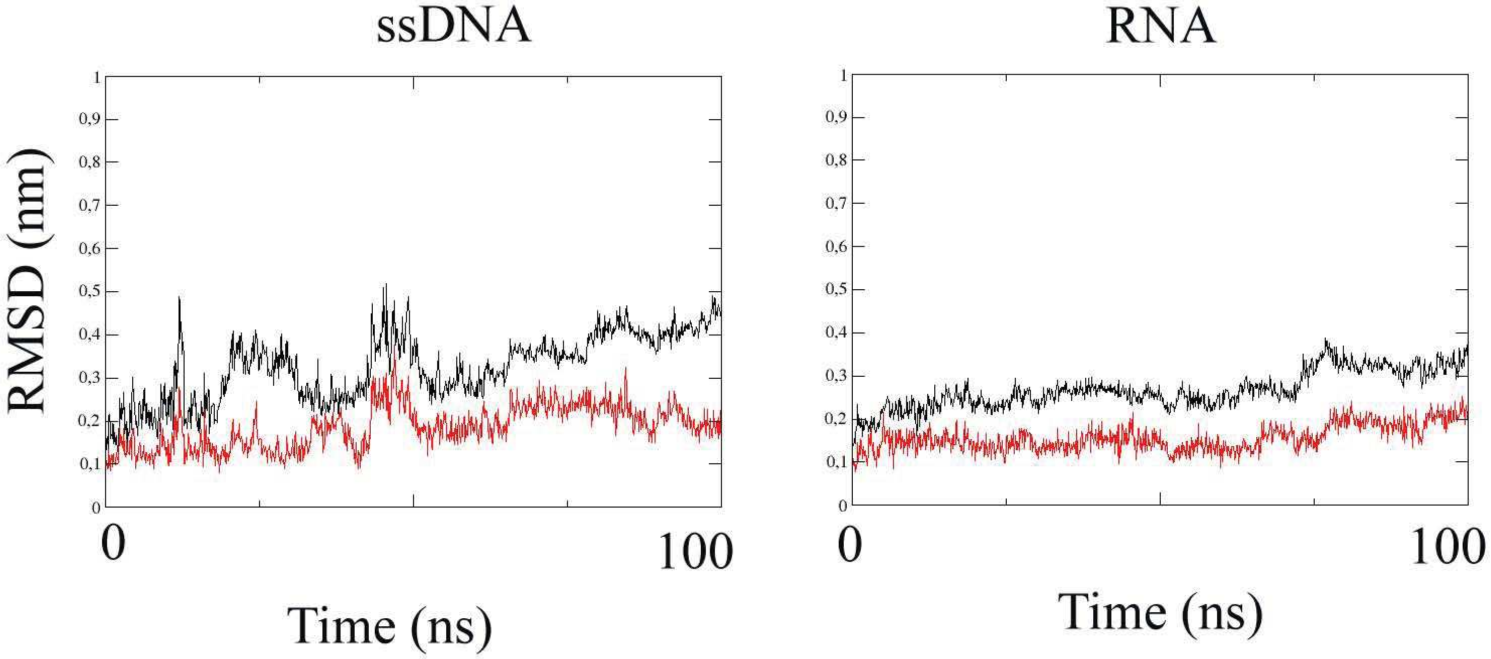


Figure S5

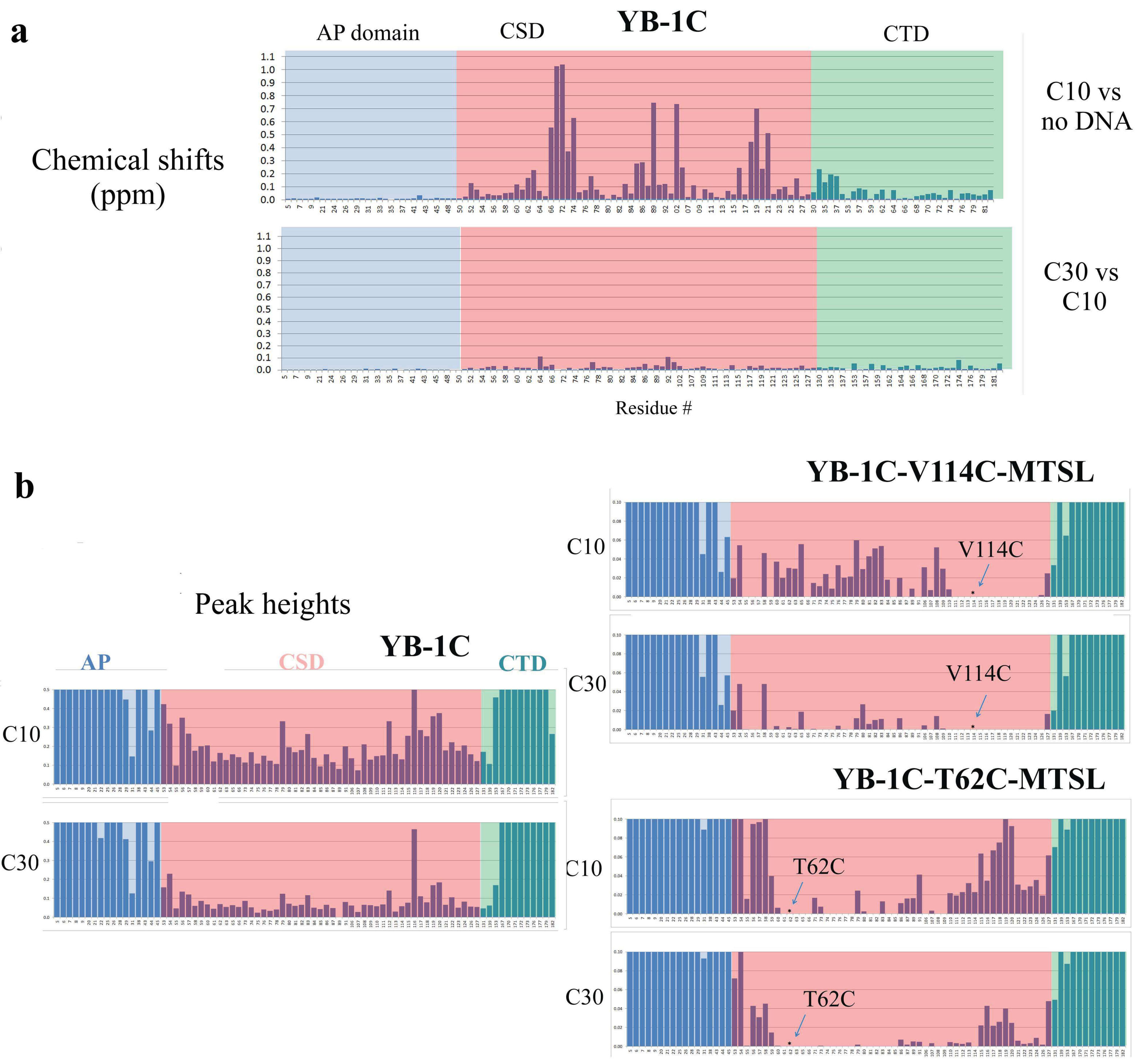
a
YB-1C
AP domain
CSD
CTD
Chemical shifts (ppm)
C10 vs no DNA
C30 vs C10
Residue #
b
Peak heights
YB-1C
AP
CSD
CTD
C10
C30
YB-1C-V114C-MTSL
V114C
YB-1C-T62C-MTSL
T62C

Figure S6

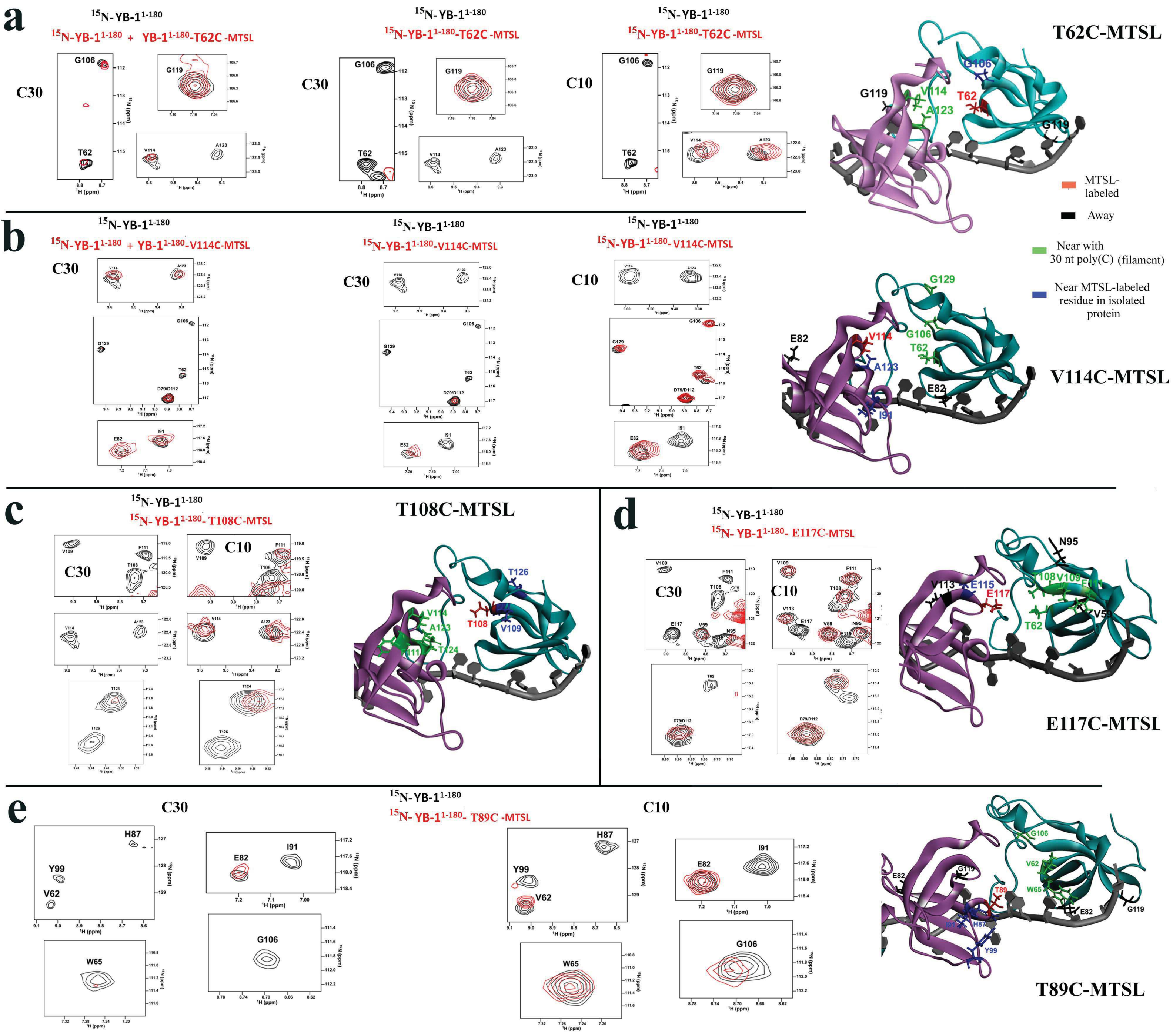
a
$^{15}$N-YB-1$^{1-180}$
$^{15}$N-YB-1$^{1-180}$ + YB-1$^{1-180}$-T62C-MTSL
C30
C10
T62C-MTSL
MTSL-labeled
Away
Near with 30 nt poly(C) (filament)
Near MTSL-labeled residue in isolated protein
b
$^{15}$N-YB-1$^{1-180}$ + YB-1$^{1-180}$-V114C-MTSL
V114C-MTSL
c
$^{15}$N-YB-1$^{1-180}$-T108C-MTSL
T108C-MTSL
d
$^{15}$N-YB-1$^{1-180}$-E117C-MTSL
E117C-MTSL
e
$^{15}$N-YB-1$^{1-180}$-T89C-MTSL
T89C-MTSL

Figure S7

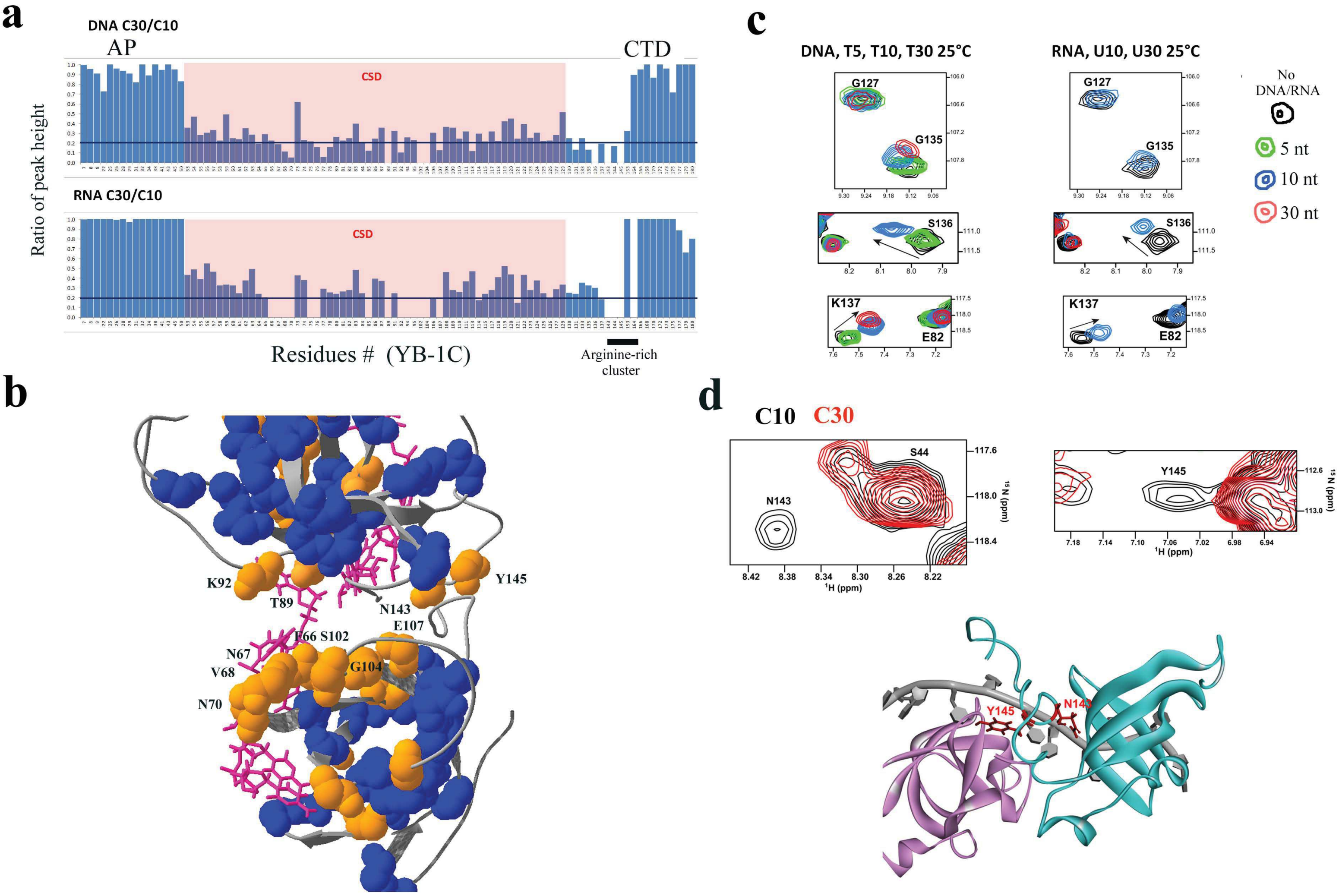
a
DNA C30/C10
AP
CTD
CSD
RNA C30/C10
CSD
Ratio of peak height
Residues # (YB-1C)
Arginine-rich cluster
b
K92
T89
N143
Y145
E107
F66 S102
N67
V68
G104
N70
c
DNA, T5, T10, T30 25°C
RNA, U10, U30 25°C
G127
G135
S136
K137
E82
No DNA/RNA
5 nt
10 nt
30 nt
d
C10
C30
S44
N143
Y145
$^{1}$H (ppm)
$^{15}$N (ppm)

Figure S8

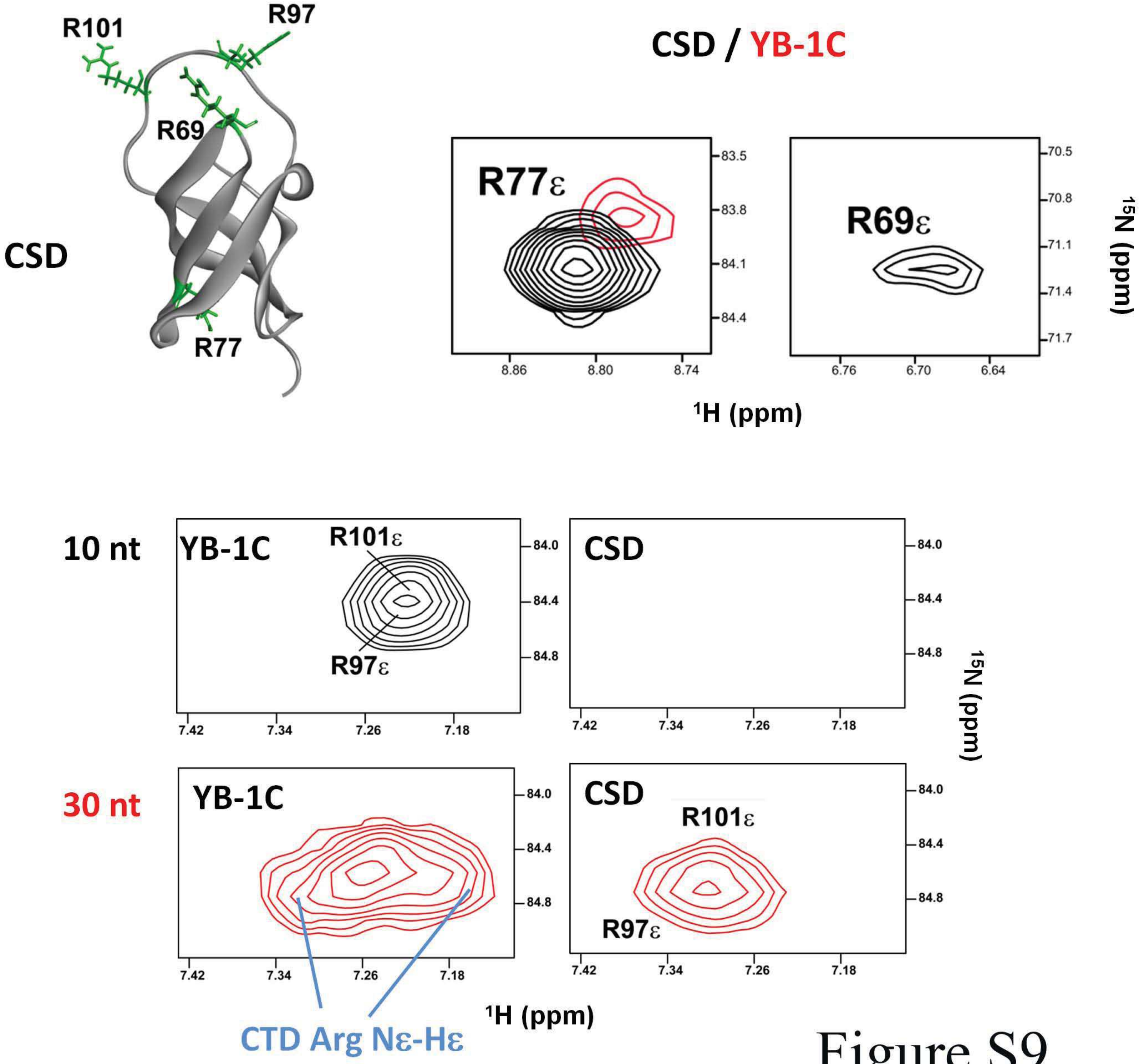
R101
R97
R69
R77
CSD
CSD / YB-1C
R77ε
R69ε
$^{1}$H (ppm)
$^{15}$N (ppm)
10 nt
YB-1C
R101ε
R97ε
CSD
30 nt
YB-1C
CSD
R101ε
R97ε
CTD Arg Nε-Hε
$^{1}$H (ppm)
$^{15}$N (ppm)

Figure S9

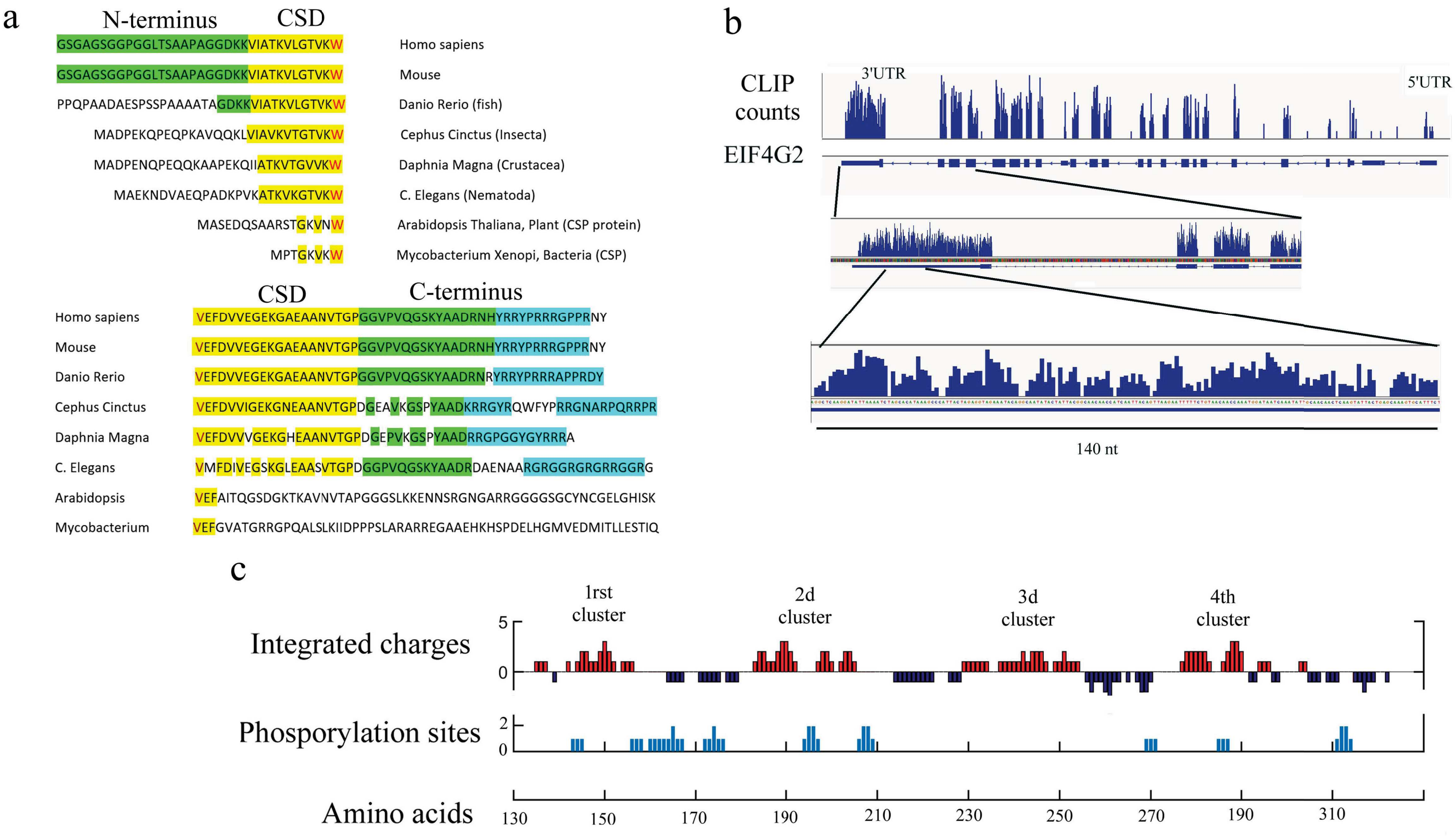
a
N-terminus
CSD
GSGAGSGGPGGLTSAAPAGGDKKVIATKVLGTVKW
Homo sapiens
GSGAGSGGPGGLTSAAPAGGDKKVIATKVLGTVKW
Mouse
PPQPAADAESPSSPAAAATAGDKKVIATKVLGTVKW
Danio Rerio (fish)
MADPEKQPEQPKAVQQKLVIAVKVTGTVKW
Cephus Cinctus (Insecta)
MADPENQPEQQKAAPEKQIIATKVTGVVKW
Daphnia Magna (Crustacea)
MAEKNDVAEQPADKPVKATKVKGTVKW
C. Elegans (Nematoda)
MASEDQSAARSTGKVNW
Arabidopsis Thaliana, Plant (CSP protein)
MPTGKVKW
Mycobacterium Xenopi, Bacteria (CSP)
CSD
C-terminus
Homo sapiens
VEFDVVEGEKGAEAANVTGPGGVPVQGSKYAADRNHYRRYPRRRGPPRNY
Mouse
VEFDVVEGEKGAEAANVTGPGGVPVQGSKYAADRNHYRRYPRRRGPPRNY
Danio Rerio
VEFDVVEGEKGAEAANVTGPGGVPVQGSKYAADRNRYRRYPRRRAPPRDY
Cephus Cinctus
VEFDVVIGEKGNEAANVTGPDGEAVKGSPYAADKRRGYRQWFYPRRGNARPQRRPR
Daphnia Magna
VEFDVVVGEKGHEAANVTGPDGEPVKGSPYAADRRGPGGYGYRRRA
C. Elegans
VMFDIVEGSKGLEAASVTGPDGGPVQGSKYAADRDAENAARGRGGRGRGRRGGRG
Arabidopsis
VEFAITQGSDGKTKAVNVTAPGGGSLKKENNSRGNGARRGGGGSGCYNCGELGHISK
Mycobacterium
VEFGVATGRRGPQALSLKIIDPPPSLARARREGAAEHKHSPDELHGMVEDMITLLESTIQ
b
CLIP
counts
3'UTR
5'UTR
EIF4G2
140 nt
c
1rst
cluster
2d
cluster
3d
cluster
4th
cluster
Integrated charges
5
0
Phosporylation sites
2
0
Amino acids
130
150
170
190
210
230
250
270
190
310

# Figure S10
